# Exosomes Derived From Heat Stroke Cases Carry miRNAs Associated With Inflammation and Coagulation Cascade

**DOI:** 10.3389/fimmu.2021.624753

**Published:** 2021-06-22

**Authors:** Yue Li, Qiang Wen, Huaisheng Chen, Xinhui Wu, Bin Liu, Hui Li, Lei Su, Huasheng Tong

**Affiliations:** ^1^ Department of Intensive Care Unit, General Hospital of Southern Theatre Command of PLA, Guangzhou, China; ^2^ Department of Critical Care Medicine, Shenzhen People’s Hospital, Second Clinical Medical College of Jinan University, First Affiliated Hospital of Southern University of Science and Technology, Shenzhen, China

**Keywords:** heat stroke, exosome, miRNA, next-generation sequencing, inflammation, coagulation

## Abstract

The pathological mechanism underlying heat stroke (HS) is associated with the dysbalanced inflammation and coagulation cascade. Cell-derived circulating extracellular vesicles (EVs), as a novel pathway mediating intercellular communication, are associated with the immune response and inflammation in critical inflammatory syndromes, such as sepsis. Although these vesicles contain genetic material correlated with their biological function, their molecular cargo during HS remains unknown. In this study, we evaluate the presence of microRNAs (miRNAs) and messenger RNAs (mRNAs) associated with inflammatory responses and coagulation cascade in exosomes of patients with HS. Blood samples were collected from three patients with HS at the time of admission to the intensive care unit; three healthy volunteers were selected as control. Exosomes were isolated using ultracentrifugation, and their miRNA content was profiled using next-generation sequencing; mRNA content was evaluated using qPCR array. Compared with those from healthy volunteers, exosomes from patients with HS showed substantial changes in the expression of 202 exosomal miRNAs (154 upregulated and 48 downregulated miRNAs). The most upregulated miRNAs included miR-511-3p, miR-122-5p, miR-155-3p, miR-1290, and let7-5p, whereas the most downregulated ones included miR-150-3p, 146a-5p, and 151a-3p. Gene ontology enrichment of the miRNAs of patients with HS compared with control subjects were associated mostly with inflammatory response, including T cell activation, B cell receptor signaling, dendritic cell chemotaxis and leukocyte migration, and platelet activation and blood coagulation. The identified miRNAs were primarily enriched to the signal transduction pathways namely, T cell receptor signaling, Ras signaling, chemokine signaling, platelet activation, and leukocyte transendothelial migration, all of which are associated with inflammation and hemostasis. Multiple targeted mRNAs associated with the inflammatory response, blood coagulation, and platelet activation were further verified in serum exosomes. Exosomes from patients with HS convey miRNAs and mRNAs associated with pathogenic pathways, including inflammatory response and coagulation cascade. Exosomes may represent a novel mechanism for intercellular communication during HS.

## Introduction

Heatstroke (HS) is a severe condition with limited treatment options that can even be fatal. Recently, HS incidence has increased due to the growing frequency of heatwaves; the resultant mortality rates have also increased, particularly HS-induced multiple organ failure, with a mortality rate of 40–60% (1, 2).

The molecular mechanism underlying HS pathogenesis is complicated and poorly investigated (3). Evidence has suggested that the HS mechanism is associated with dysbalanced inflammation and coagulation cascade (4). Cell-derived circulating extracellular vesicles (EVs) shed from different cell types during acute inflammatory syndromes such as sepsis. The roles of EVs in disease pathophysiology have been extensively investigated, especially those associated with immune responses and inflammation (5, 6). In addition, we have previously demonstrated that hepatocyte-derived EVs from patients with HS correlate with liver dysfunction, which is associated with activation of EV-induced inflammatory NOD-like receptor pathway and necrosis in hepatocytes (7, 8).

EVs can potentially mediate cell communication under normal physical conditions or disease state *via* functional microRNAs (miRNAs) and messenger RNA (mRNA). A pilot study has suggested, using pathway enrichment analysis, that the miRNA profile in serum EVs from sepsis patients might have had significant roles during sepsis (9). The authors also reported that the differential expression pattern of EV-miRNA in sepsis modulates the immune system and cell cycle. However, no existing study has yet examined the genetic exosomal content in HS.

This study analyzes the changes in the miRNA profiles in serum EVs and their potential biological functions in patients with HS. The results of this study provide insights into the pathophysiology of HS and the development of novel treatment strategies.

## Materials and Methods

### Patient Recruitment and Clinical Data Collection

From June 2019 to June 2020, six patients admitted within the first 24 h of severe HS onset into the ICU at the General Hospital of South Theatre of People’s Liberation Army (PLA) of China, a tertiary hospital, were retrospectively enrolled in this study. HS was diagnosed according to the Expert Consensus on Diagnosis and Treatment of Heat Stroke in China (10) released by the Expert Group on Prevention and Treatment of Heat Stroke and Critical Care Committee of PLA of China. Patients with active malignant tumors, chronic liver and kidney diseases, chronic cardiac insufficiency (New York grades 3–4), chronic pulmonary insufficiency, underlying central nervous system (CNS) disease, metabolic disorders, or those using heparin or any other medications, were eliminated from this study. Additionally, three healthy individuals from the Physical Examination Center were enrolled as the control group. Baseline demographic characteristics were recorded upon admission to ICU (day 1). Blood samples and organ function data were collected on day 1; besides, the sequential organ failure assessment (SOFA) and the age and chronic health evaluation (APACHE II) scores were determined to evaluate the disease severity. Patients or the corresponding family members provided informed consent. Our study protocol was approved by the Medical Ethics Review Committee of General Hospital of Southern Theater of PLA of China.

### Blood Sample Collection and Exosome Isolation

A total of 30 ml peripheral venous blood sample was collected from each patient using the ethylenediaminetetraacetic acid (EDTA) anticoagulant tubes (the first few 5 ml was discarded). Next, the blood collection tubes were left standing vertically for 30 min at 22–27°C. The plasma was obtained from whole blood following centrifugation for 10 min at 4°C and 2,500 × *g*. After the addition of protease inhibitors (supplemented with 3 mM phenylmethylsulfonyl fluoride, 1 µg/ml pepstatin, as well as 1 µg/ml aprotinin), the plasma was stored at −80°C until further use. When processing, plasma was diluted with equivalent volume of phosphate-buffered saline (PBS) and centrifuged at 4°C (30 min at 2,500 × *g*, 45 min at 12,000 × *g*, and 2 h at 110,000 × *g*, SW 28 Ti Rotor, Optima L-90K Ultracentrifuge, Beckman Coulter, Fullerton, USA). Finally, 50–200 µl PBS was used to resuspend the precipitate (exosomes) and was stored in −80°C.

### Exosome Morphology Observed *via* Transmission Electron Microscopy

Exosome samples were collected from a healthy individual, a patient with mild HS, and a patient with severe HS (randomly selected) and prepared for TEM evaluation. Firstly, exosomes were dehydrated in 2% formalin. After adding 5 ml exosome suspension into the copper grid coated with formvar (Mecalab, QC, Canada), the sample was incubated for 30 min, at 4°C, washed with 100 µl PBS, fixed with 2% paraformaldehyde for 10 min, and stained for 15 min with 2% uranyl acetate (dissolved into 50% ethanol). Thereafter, the Philips CM10 transmission electron microscope (JEM-2100F, Netherlands) was utilized for sample visualization.

### Nanoparticle Tracking Analysis

For detecting exosome content and size distribution, NTA was performed using the NanoSight NS3000 (Malvern Instruments, Worcestershire, UK). In brief, exosome sample was diluted with sterile PBS at 1:5,000, and the NanoSight LM10 and NTA software (NanoSight Ltd, Amesbury, UK) was employed for sample analysis, thrice (60 s each).

### Western Blotting

Proteins were extracted from 1 ml ultracentrifuged plasma (120,000 × *g* for 2.5 h) using the DE buffer (consisting of 20 mM Tris-HCL, 12 mM 2-mercaptoethanol, 1 mM EGTA, 1 mM EDTA, 1% Triton-X 100, together with 10% glycerol) that contained the protease inhibitor mix (GE Healthcare, Uppsala, Sweden). Total proteins were quantified using Bradford, then 5 µg protein from each sample was separated on a 12% SDS-PAGE. The protein bands were then transferred onto nitrocellulose membranes and blotted using polyclonal antibodies against CD9 (1:1,000 diluted, Abcam ab10895, Cambridge, UK), CD63 (1:1,000 diluted, Abcam ab92726, Cambridge, UK), and Tsg-101 (1:1,000 diluted, Abcam ab30871, Cambridge, UK) at 4°C, overnight. GAPDH was used as a loading control. The target protein expression level was quantified by densitometry analysis using the ImageJ software.

### Exosomal RNA Sequencing (Next-Generation Sequencing)

#### Exosomal RNA Extraction

TRIzol reagent was used to extract the RNA from exosomes.

#### Library Construction and Sequencing

The NEB SmallRNA Library kit was adopted for library construction, whereas Illumina HiSeq was applied for sequencing, with a data volume of 20 M reads per sample.

#### Filtering and miRNA Mapping

Clean data were filtered from raw reads after sequencing in accordance with the following criteria: a) 30% base quality <20; b) read length <17 bp; c) adaptor sequence. Clean data were then mapped to the Danio Rerio miRNA database (miRBase v21.0) together with the Danio Rerio genome (Zv10, NCBI) using the BWA software, whereas the unmapped reads were used to map the rat, mouse, and human miRNA database (miRBase v21.0) to achieve the expression profiles of miRNAs.

#### New miRNA Prediction

The novel miRNA prediction was applied using mirdeep based on the reads mapped to Zv10 genome but unmapped against the Danio Rerio miRNA database (miRBase v21.0) to identify new miRNA. The BWA software mapping was applied in measuring the expression of new miRNA (11).

#### Mapping of RNA Sequencing

Pair-end read mapping. Before read mapping, adaptor sequences, low-quality reads with over 20% bases that had <20 quality and those having over 5% ambiguous bases (N) were eliminated from raw reads to obtain clean reads. The zebrafish genome (version: Zv10) was used to align clean reads by the Tophat program (12).

#### Differential-Gene-Finder

The DESeq2 package was adopted to identify miRNA and mRNA with differential expression according to the numbers of miRNA and mRNA, FDR, and P-value (13), with the following selection criteria: i) Fold-Change (FC) >2 or <0.5; ii) P-value <0.05 and FDR <0.05.

#### Target Analysis

Miranda target analysis (14) was adopted for predicting the miRNA target in the mRNA of zebrafish. To identify the possible miRNA target, targets with no negative association of mRNA with miRNA were excluded.

### Gene Ontology Functional Annotations

GO analysis was conducted to identify the roles of specific genes in typical or significant differentially expressed gene (DEG) profiles (15). The GO annotations were first downloaded from Gene Ontology (http://www.geneontology.org/), UniProt (http://www.uniprot.org/), along with NCBI (http://www.ncbi.nlm.nih.gov/) databases. Thereafter, significant GO categories were identified using Fisher’s exact test, whereas P-values were corrected using FDR.

### Pathway Enrichment Analysis

Pathway enrichment analysis was also applied to identify DEGs based on the Kyoto Encyclopedia of Genes and Genomes (KEGG) database. Significant pathways were selected using Fisher’s exact test, with FDR and P-value satisfying significance thresholds (16).

### qPCR Data Analysis

The Statminer v5 software (Integromics, Granada, Spain) was used to analyze the cycle threshold (Ct) data. According to the manufacturer’s instructions, Ct values ≥32 were excluded from immune and miRNA studies. For the OS studies, the cutoff value was set at 35 in line with the manufacturer’s instructions. For the miRNA analysis, the optimal reference was identified using the geNorm approach, and the median miR17, miR20a, and miR106a were applied to normalize the data. The 18S rRNA was used to normalize the immune gene expression, and beta-actin was used to normalize OS gene expression. Finally, the 2^DDCt^ method was adopted to calculate the relative gene expression.

### Statistical Analysis

The data normality was assessed using the Kurtosis test. Data with normal distribution were expressed as mean ± standard deviation (SD), whereas those with abnormal distribution were expressed as median ± interquartile range. The two-tailed Student’s *t*-test was used to compare two groups, whereas one-way analysis of variance followed by *post hoc* test was used to compare multiple groups. A P*-*value of <0.05 (two-tailed) was considered statistically significant, whereas GraphPad version 7.0 (La Jolla, CA, USA) was used for all tests.

## Results

### Patients Baseline Characteristics and Outcomes

All patients and healthy controls were males with low comorbidities. Ages were comparable between healthy controls and patients with HS (25 ± 6 *vs.* 21 ± 4 years, respectively). No participant has previously used any medication. Clinical and laboratory data are summarized in **Table 1**. Core body temperatures on admission were higher in patients with HS (39.00 ± 1.94 *vs.* 36.42 ± 0.46°C, P < 0.001). In addition, three out of the six patients with HS had died during the ICU hospitalization. The patients remained at ICU for 5–10 (median, 7) days.

**Table 1:** Basic clinical characteristics and disease severity scores of the participants.

Characteristics	Healthy controls (n = 3)	Severe HS (n = 6)	P-value
ICU length of stay (days), median (interquartile range)	0 (0)	10 (3–17.45)	<0.001
T (°C)	36.42 ± 0.46	39.00 ± 1.94	<0.001
APACHE II score	0.47 ± 0.83	17.05 ± 4.35	<0.001
SOFA score	0.6 ± 0.63	10.95 ± 5.64	<0.001

APACHE, Acute Physiology and Chronic Health Evaluation; ICU, intensive care unit; SOFA, Sequential Organ Failure Assessment; T, body temperature.

All severe HS cases were complicated with multiple organ dysfunction. There was significant organ support when patients were enrolled because 67% of patients received mechanical ventilation, whereas 50% received norepinephrine. The overall disease severity, as assessed using the APACHE II and SOFA scores, was 17.05 ± 4.35 and 10.95 ± 5.64, respectively (**Table 2**). None of the enrolled patients received prophylactic heparin, or transfusion of red blood cells/platelet/fresh frozen plasma, or antibiotics on admission before the collection of study samples.

**Table 2:** Comparison of clinical and laboratory indices of patients with heat stroke and healthy controls according to the day of admission and outcome status.

Characteristics	Healthy controls (n = 3)	Severe HS (n = 6)	P-value
Hemodynamic data			
HR (beats/min)	74.3 ± 7.74	96.85 ± 32.43	<0.001
MAP (mmHg)	77.6 ± 6.99	72.15 ± 20.95	0.782
Vasoactive drug, n (%)	0 (0)	3 (50)	<0.001
Lactate (μmol/L)	1.07 ± 0.47	3.11 ± 2.55	<0.001
Ventilatory data			
PaO2/FiO2	378.7 ± 72.25	312.3 ± 67.56	0.007
MV, n (%)	0 (0)	6 (67)	<0.001
Inflammatory data			
WBC (×109 cells/L)	9.42 ± 3.14	10.81 ± 4.47	<0.001
PCT (ng/ml)	0.34 ± 0.34	4.06 ± 4.14	<0.001
Hepatic data			
ALT (U/L)	25.51 ± 13.7	1,448 ± 2,360	<0.001
AST (U/L)	22.4 ± 13.8	2,144 ± 3,861	0.001
TBil (µmol/L)	9.23 ± 4.45	62.83 ± 93.67	<0.001
ALB (g/L)	40.79 ± 5.36	37.56 ± 3.51	0.302
Renal data			
Cr (μmol/L)	95.4 ± 27.28	161.1 ± 84.24	<0.001
BUN (mmol/L)	5.51 ± 2.19	8.03 ± 6.73	<0.001
Urine output (ml/d)	2,680 ± 727.2	2,095 ± 1,369	<0.001
Coagulation data			
PT (s)	13.39 ± 0.93	25.67 ± 15.61	<0.001
INR	13.39 ± 0.93	2.50 ± 2.21	<0.001
Fib (g/L)	3.53 ± 0.66	2.05 ± 0.76	< 0.001
PLT (×109/L)	219.6 ± 65.05	101.2 ± 61.35	< 0.001
D-dimer	1.46 ± 1.32	15.77 ± 5.96	< 0.001
FDP	6.83 ± 2.88	100.8 ± 184.3	0.001
Rhabdomyo data			
CK (μg/L)	54.33 ± 23.52	5,155 ± 5,888	<0.001
MYO (μg/L)	48.15 ± 24.91	1,374 ± 964.4	<0.001
Cardiac data			
CK-MB	2.54 ± 1.45	39.83 ± 50.51	<0.001
cTnI	12.76 ± 9.94	874.2 ± 1134	0.02
CNS data			
GCS score	15 ± 0	8.3 ± 4.28	<0.001

ALB, albumin; ALT, alanine aminotransferase; AST, aspartate aminotransferase; BUN, blood urea nitrogen; CK, creatine kinase; CK-MB, CK-myocardial band; CNS, central nervous system; Cr, creatinine; cTnI, cardiac troponin I; FDP, fibrin degradation product; Fib, fibrin; FiO_2_, percentage of inspired oxygen; GCS, Glasgow Coma Scale; HR, heart rate; INR, international normalized ratio; MAP, mean arterial pressure; MV, mechanical ventilation; MYO, myoglobin; PaO_2_, partial pressure of arterial oxygen; PCT, procalcitonin; PLT, platelet; PT, prothrombin time; TBil, total bilirubin; WBC, white blood cell.

### Characterization of Plasma Exosomes

**Figure 1:**
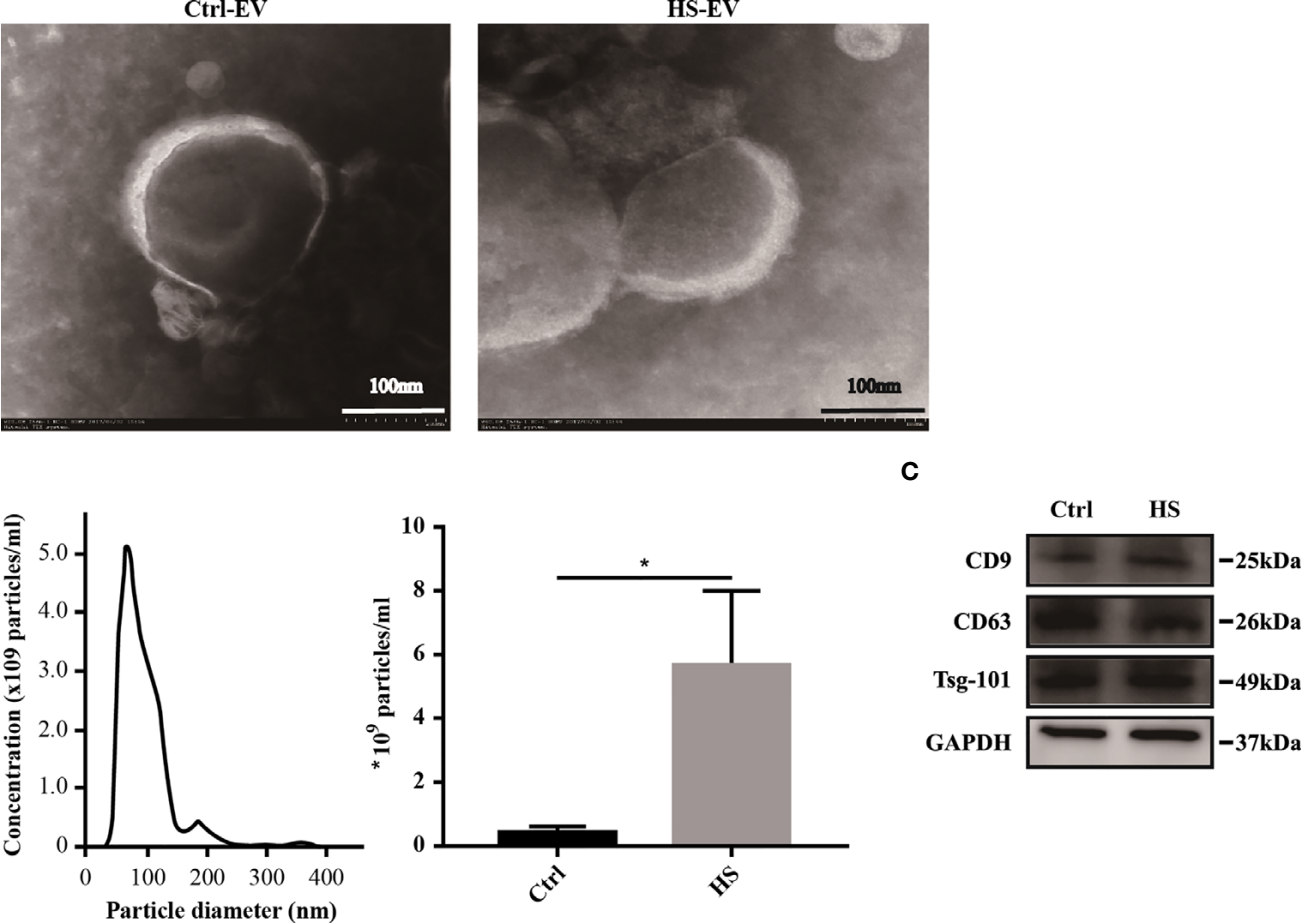
Characterization of plasma exosomes in both healthy controls and HS patients. **(A)** Morphology of plasma exosomes visualized under TEM. Bar, 100 nm. **(B)** Exosome size distribution examined through NTA. (*) *p* < 0.05. **(C)** Levels of representative exosomal surface markers (CD9, CD63 along with Tsg-101) using western blot analysis. All experiments were repeated thrice.

TEM showed that the separated preparation from the plasma of healthy controls and patients with HS exhibited a double-membrane vesicle-like structure approximately 100 nm in diameter (**Figure 1A**). Besides, the NTA results indicated that the plasma exosome concentration of the HS group increased compared with that of the control group (5.67 ± 2.34 × 10^9^ *vs.* 0.47 ± 0.15 × 10^9^ particles/ml, respectively; **Figure 1B**). Finally, western blotting analysis showed the positive expression of the membrane-associated proteins related to endocytosis, as well as the exosomal markers (CD9, CD63, and Tsg-101) in both control and HS groups (**Figure 1C**). Taken together, the extracted vesicles were mostly consistent with exosomes.

### HS Significantly Changed the Expression Patterns of Circulating Exosomal miRNAs

**Figure 2:**
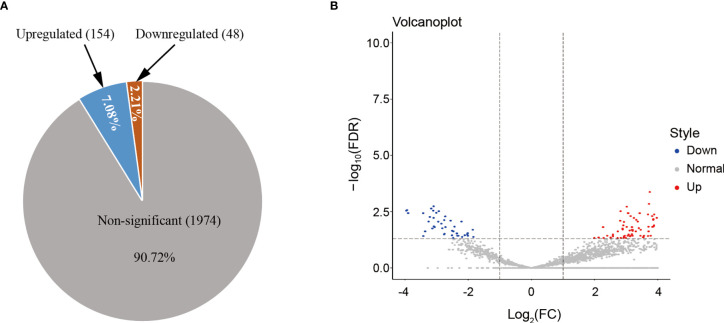
**(A)** HS altered the miRNA expression patterns of hepatocyte-derived EVs, as analyzed using NGS. The number and proportion of differentially regulated (upregulated, downregulated, and non-significant) EV miRNAs isolated from HS compared with the control (n = 3 samples/group). Numbers indicate proportions. Data base: Homo Sapiens. **(B)** Volcano plot representing the miRNA fold-change and RNA intensity in the HS and control hepatocyte EVs. X-axis shows miRNA fold-change (HS/Control, log2), whereas Y-axis depicted the summary of RNA intensity (LFQ intensity, lg).

To determine whether HS changed exosomal miRNA expression, the miRNA profiling analysis was conducted firstly among the HS cases admitted to ICU, and the results were compared against the healthy controls. HS had no impact on 90.72% of miRNA accumulation (**Figure 2A**). **Figure 2B** shows that differences in most identified miRNAs were not significant due to the close abundance ratio of HS *versus* control EVs. Nonetheless, in patients with HS, 202 miRNAs (9.28%) were identified as differentially expressed compared with controls [154 upregulated (7.08%) and 48 downregulated (2.21%)] (**Figure 2A**). The miRNAs expression FC in HS *versus* control EVs (ratio of HS to control group <0.5 or >2), along with the P-values are summarized in **Table 1** and displayed in **Figure 2B**.

**Figure 3:**
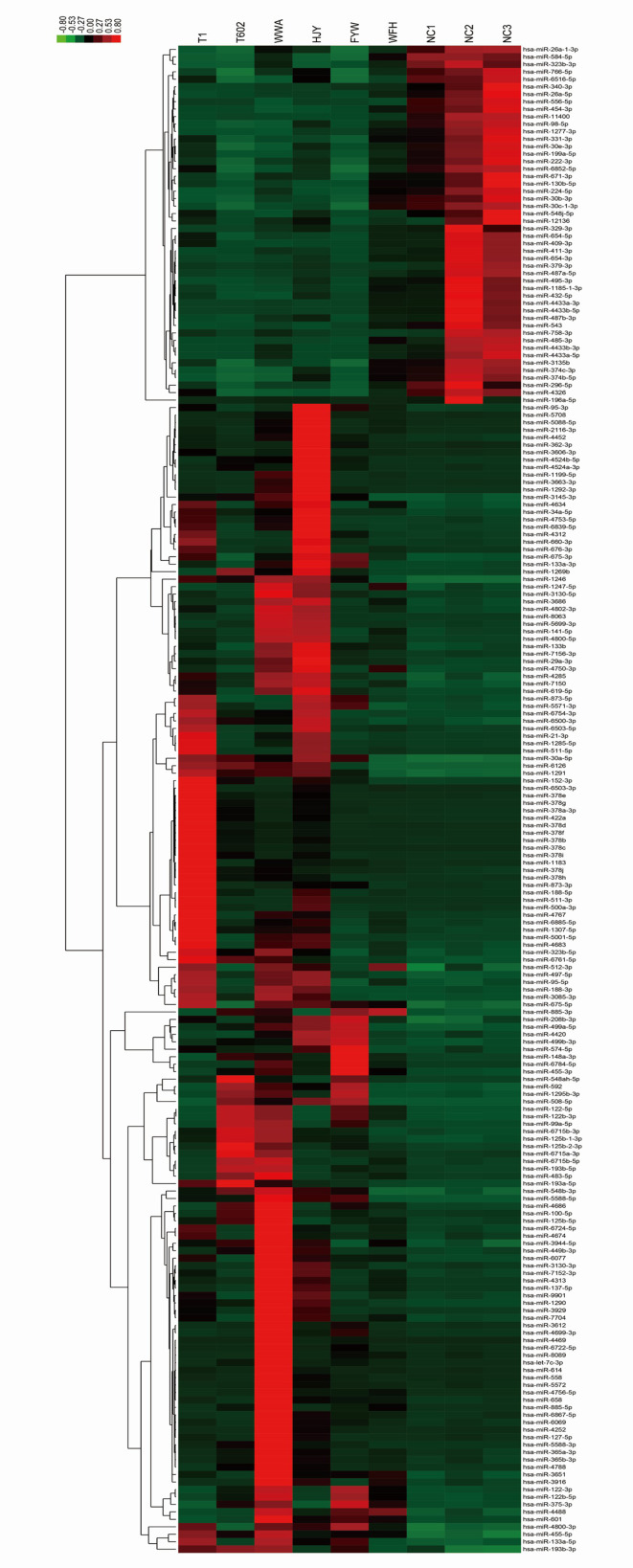
Cluster analysis of the dysregulated miRNA components from HS EVs. The list of all 202 significantly differentially expressed miRNAs (HS-EV group/control EV group) has been shown in heat map.

Additionally, hierarchical cluster analysis was conducted on 202 differentially expressed miRNAs based on samples or stimulants to examine the creditability of those selected target miRNAs (**Figure 3**). There was significant differential expression in HS patients *versus* controls, with low SD in three samples, which suggested that our results were highly repeatable. Some specific miRNAs with significant elevation within HS EVs relative to controls were identified, including miR-511-3p, miR-122-5p, miR-155-3p, miR-1290, and let7-5p. The most downregulated miRNAs included miR-150-3p, 146a-5p, and 151a-3p. The ACC IDs of all the dis-regulated miRNAs and the corresponding FCs are presented in **Table S1**. The results suggested that, HS changed certain miRNA levels within plasma EVs, and this was correlated with the biological effect.

### The Upregulated and Downregulated miRNAs in HS EVs Were Associated With the Inflammation and Coagulation Signal Transduction Pathways

Thereafter, each EV miRNA identified using NGS was classified to determine the specific biological role using GO functional annotation clustering approach. Most identified EV miRNAs were involved in regulating the signaling and molecular functions. Therefore, the targeted miRNAs may influence the EVs’ biological activity.

**Figure 4:**
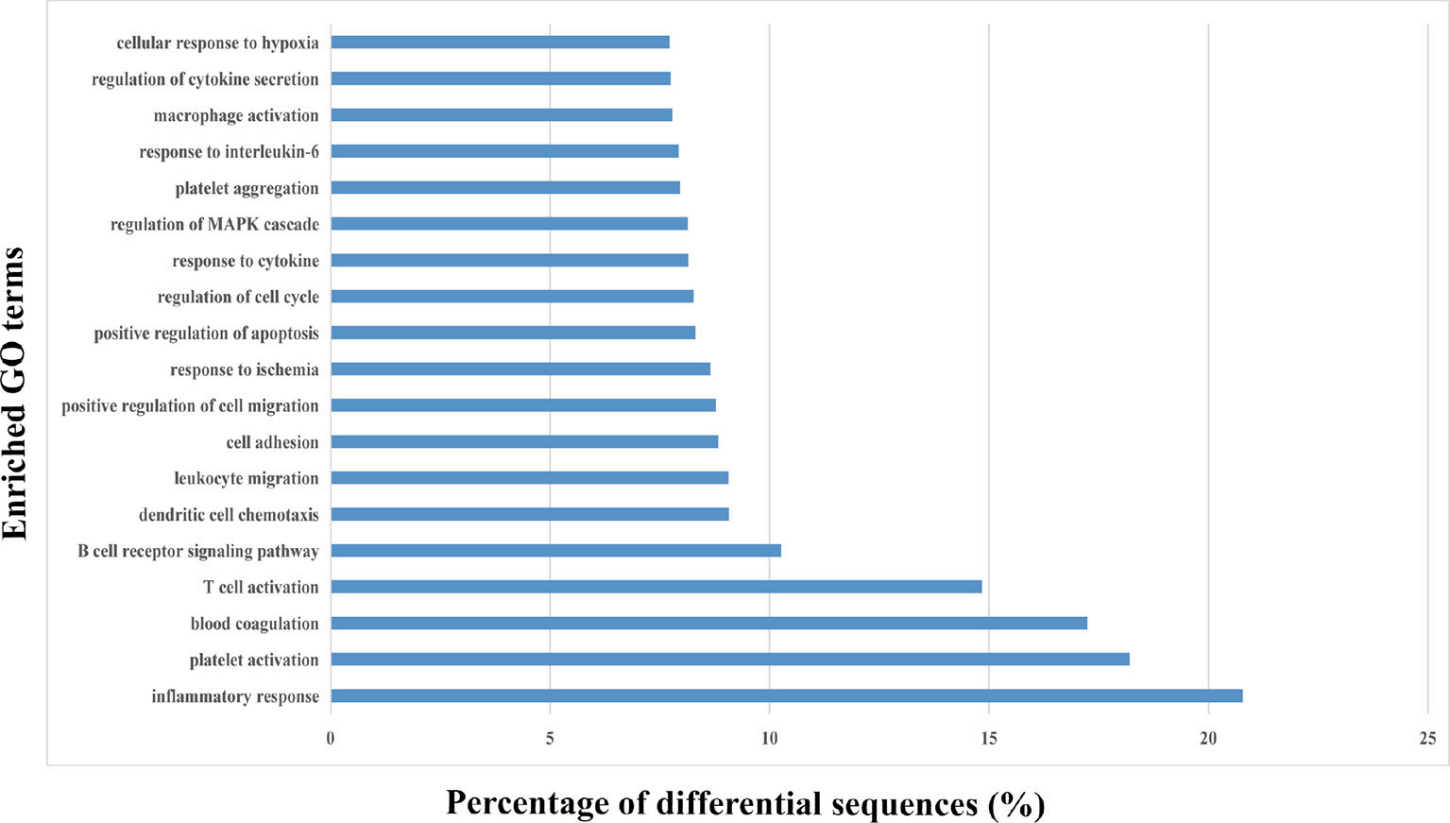
Gene ontology (GO) enrichment analysis of the differentially regulated proteins. The top 20 enriched terms according to the GO functional annotation clustering of the 202 differentially expressed miRNAs in the heat-stroked hepatocyte-derived EVs. Percentages of the sequences involved are shown.

The 20 most significant biological terms related to the upregulated or downregulated miRNAs in HS EVs, as well as the miRNA enrichment significance determined for all clusters, are illustrated in **Figure 4**. Particularly, inflammatory responses including T cell activation, B cell receptor signaling, dendritic cell chemotaxis and leukocyte migration, and platelet activation and blood coagulation, as well as cell responses to various stimuli including cytokines, regulation of cell death and cell adhesion/migration, and cell cycle were the most significantly upregulated clusters and were all involved in the mechanism of HS.

**Figure 5:**
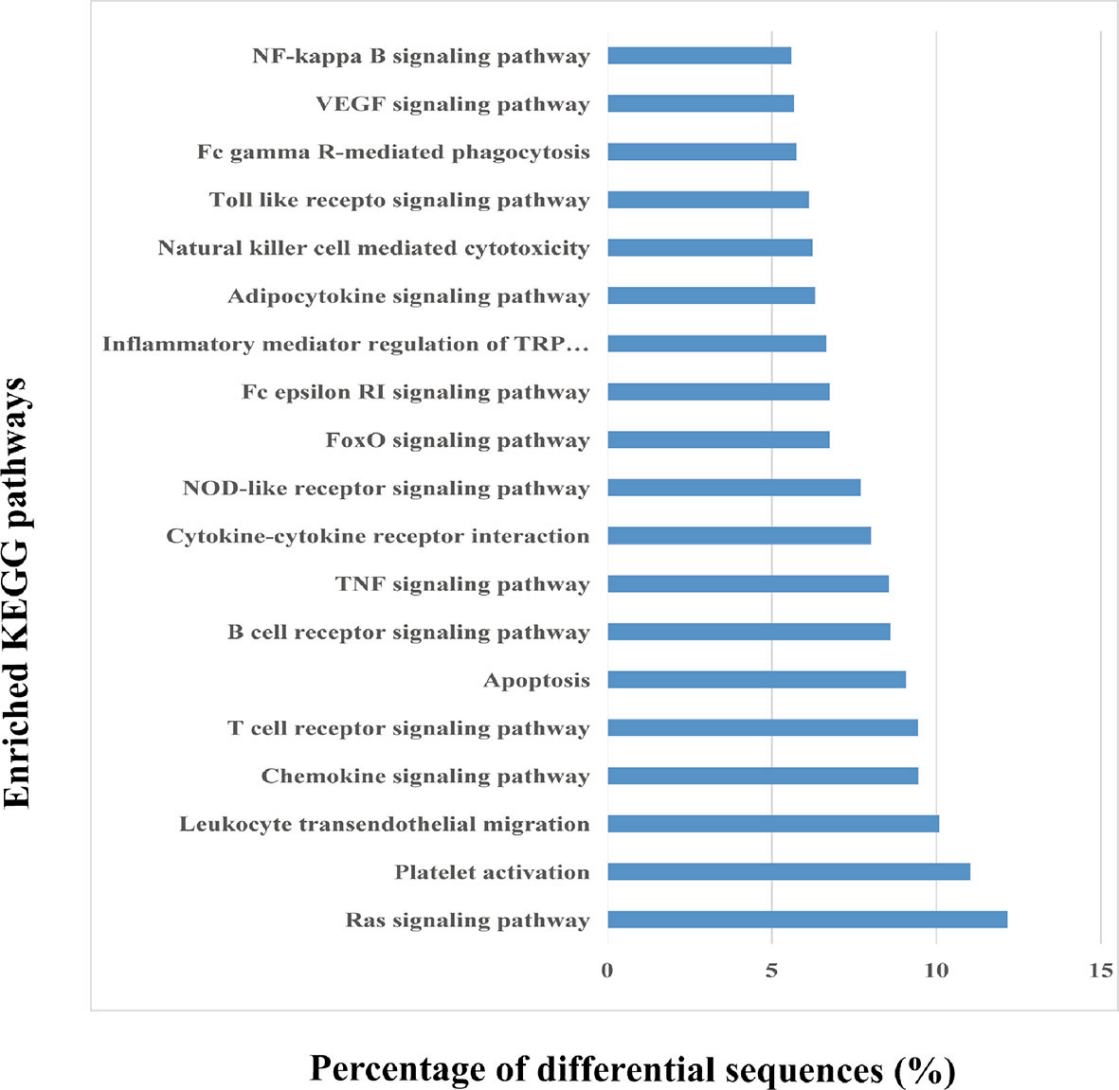
Kyoto Encyclopedia of Genes and Genomes (KEGG) pathway analysis of the differentially expressed EV miRNAs. The top 20 enriched KEGG pathways in the heat-stroked hepatocyte-derived EV-miRNA and the percentages of the sequences involved in each pathway are shown.

KEGG enrichment helps to determine the miRNA enrichment significance of every pathway. In this study, KEGG analysis indicated that those identified miRNAs were primarily enriched to the signal transduction pathways namely, T cell receptor signaling, Ras signaling, chemokine signaling, platelet activation, and leukocyte transendothelial migration (**Figure 5**). These pathways are mainly associated with inflammation and hemostasis. Thus, circulating EVs may be associated with HS-induced injury *via* the activation of these signal transduction pathways within target cells.

### Targeted mRNAs Are Associated With The Inflammatory Response, Blood Coagulation, and Platelet Activation

**Tables S2–S4** list the targeted mRNAs associated with the top three upregulated clusters, inflammatory response, blood coagulation, and platelet activation.

**Figure 6:**
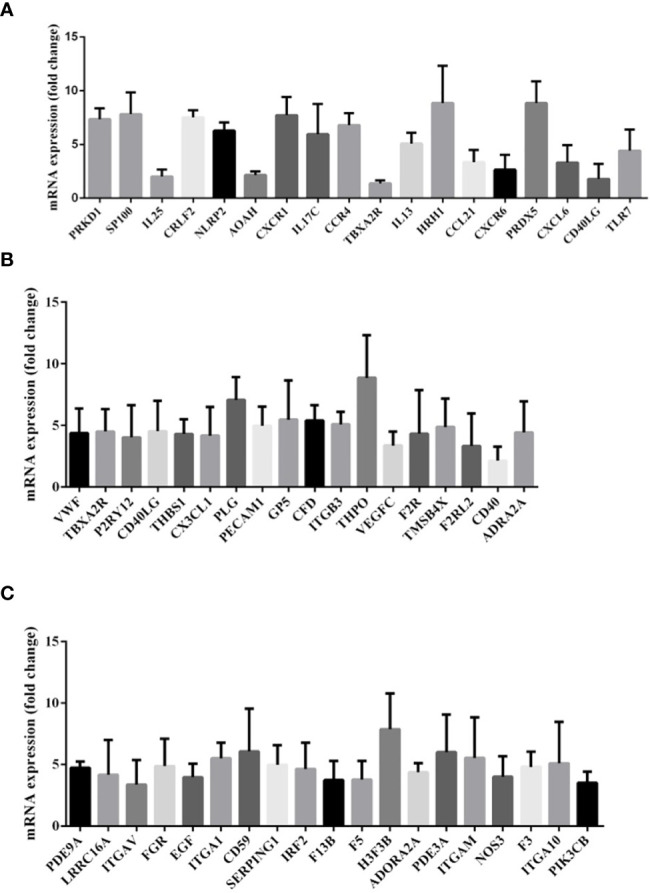
**(A)** Gene expression of messenger RNA (mRNA) related to inflammatory response in the comparison between patients with heat stroke *versus* healthy control subjects. **(B)** Gene expression of messenger RNA (mRNA) related to platelet activation in the comparison between patients with heat stroke *versus* healthy control subjects. **(C)** Gene expression of messenger RNA (mRNA) related to blood coagulation in the comparison between patients with heat stroke *versus* healthy control subjects.

For assessing the presence or absence of immune response-related mRNAs within exosomes, qPCR was performed to compare the inflammation/immune-related gene expression levels based on RNA extracted from patients with HS and healthy individuals. A total of 18 genes, including PRKD1, SP100, IL25, CRLF2, NLRP2, AOAN, and CXCR1 were significantly upregulated in patients with HS (P < 0.05; **Figure 6A**).

As illustrated in **Figure 6B**, the platelet activation-related mRNA expression levels in exosomes like VWF, TBXA2R, P2RY12, CD40LG, and THBS1 in patients with HS were also upregulated in comparison with those in healthy controls.

We also investigated the expression of mRNAs associated with blood coagulation and demonstrated an increased expression of exosomal mRNAs of PDE9A, LRRC16A, ITGAV, FCR, EGF, ITGA1, CD59, SERPING1, and IRF2 (**Figure 6C**).

**Figure 7:**
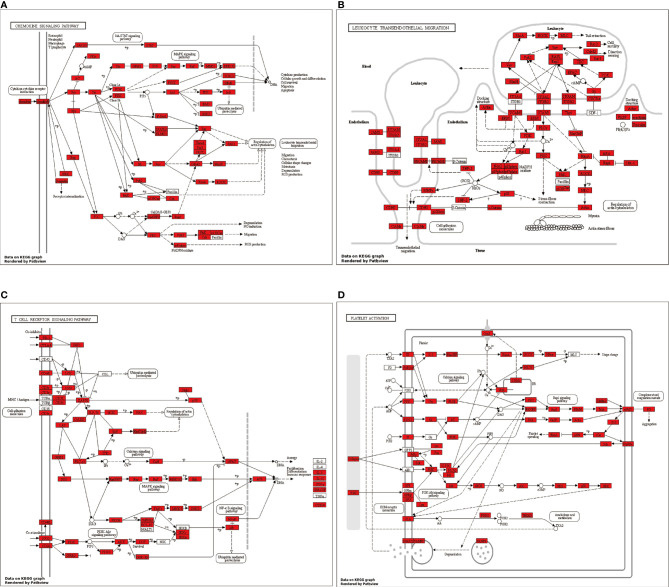
Putative **(A)** chemokine signaling pathway, **(B)** platelet activation pathway, **(C)** T cell receptor signaling pathway, and **(D)** leukocyte transendothelial migration pathway were constructed based on KEGG mapping. Red squares indicate proteins identified as differentially expressed miRNAs; white circles indicate miRNAs not identified as differentially expressed in our study.

Putative pathways, including chemokine signaling, platelet activation, T cell receptor signaling, and leukocyte transendothelial migration, were constructed based on KEGG mapping (**Figure 7**).

## Discussion

In the present study, the different circulating exosome-miRNA expression profiles with the corresponding targets involved in the immune and coagulation system were firstly identified in patients with HS.

The organ injury mechanism during HS remains unknown. The current consensus is that an inflammatory and coagulation response initiated by the hyperthermal injury may play a more important role than the direct physical damage from heat exposure (3). The pathological findings of HS (core temperature attained 42.5–43°C) baboon models include massive transmural leukocyte migration, widespread microthrombosis, microvascular endothelium injury, endothelial leukocyte–platelet interaction, and extensive apoptosis in multiple organs including the spleen, gut, liver, kidney, and lung (4).

As demonstrated in the present study, the enriched miRNAs conveyed in circulating exosomes may have participated in the inflammatory response and coagulation cascade, suggesting that exosomal miRNAs may potentially mediate HS-associated damage. Data on the presence of miRNAs in exosomes during HS are limited. We have previously studied exosomes derived from human vascular endothelial cells exposed to 41°C hyperthermal stress (17). Gene expression profiles in the lung tissue of an HS rat model showed that the miRNAs associated with inflammatory/immune responses, including leukocyte migration, lipopolysaccharide response, NIK/NF-kappaB signaling, and response to reactive oxygen species, were upregulated, which is in agreement with our findings (18). Whole blood mRNA and microRNA have been identified as biomarkers of tissue damage and immune function resulting from HS (19).

In other acute critical inflammatory diseases, miRNAs are involved in the immune/inflammatory responses during sepsis (20). In one study, miRNA profiling analysis on ten subjects with polytrauma, sepsis, and septic shock identified specific differentially expressed miRNAs (21). Furthermore, Real et al. (9) enrolled 24 patients with septic shock and reported that the circulating exosomal miRNAs were significantly altered, and the same expression kinetics was maintained during the course of the disease. These results suggest similar physiological responses as those in severe inflammatory injury.

miR-150 is one of the earliest detected miRNAs in critically ill patients or those with sepsis. Through microarray gene expression profiling, miR-150 has been reported as a part of the miRNA panels with abnormal expression within leukocytes/PBMC obtained from sepsis patients compared to normal subjects (22–24). Such alterations are reflected by consistent changes in the serum miR-150 expression. For instance, Vasilescu et al. (22) discovered that miR-150 expression was downregulated in 16 cases with abdominal sepsis. The reduced miR-150 expression in serum was associated with increased SOFA scores and sepsis severity. Furthermore, Ma et al. (25) discovered that miR-150 expression was downregulated in sepsis patients compared with that with non-infectious systemic inflammatory response syndrome or normal subjects.

miRNA-122 was one of the most significantly enriched miRNAs in our profiling. Changes in its serum concentration significantly increase systemic inflammatory diseases such as sepsis, which is associated with the inflammatory response level. Therefore, miRNA-22 is a reliable biomarker for early disease stratification and prognostic evaluation (26). The level of miRNA-122 can predict the degree of inflammation and organ injury, such as acute respiratory distress syndrome (27) and coagulation disorders (28). According to the longitudinal samples obtained from sepsis patients, the miR-122 level was significantly increased on day 14 upon ICU admission, which strongly correlated with antithrombin III (R = 0.913, P < 0.001) (26). To determine the direct or indirect impacts of miRNAs on coagulation, the crosstalk between cytokines, miRNAs dysregulation, and thrombocyte synthesis/apoptosis should be further investigated.

miRNA-155 was also among the most upregulated miRNAs in our study. In a heat stress cell model, microRNA-155 promoted heat stress-induced inflammatory responses in the microglia by facilitating inflammatory factor expression by increasing NF-*κ*B pathway activation *via* targeting liver X receptor *α* (29).

Little is known regarding the interactions between exosomal miRNAs and the corresponding targets. It has been proposed that exosomes can act as effective carriers of genetic materials and proteins to the surrounding or distant, cells. Nonetheless, the exosome–recipient cell interaction mechanism remains unknown. Such interaction generally begins with the internalization of exosomes through various pathways, such as phagocytosis, clathrin-mediated endocytosis, as well as macropinocytosis (30). The exosomal miRNAs and mRNAs in recipient cells have specific functions and may interact with related targets to synthesize novel proteins or modulating gene expression levels (31). However, the interaction of exosomal genetic contents with the target cells in HS remains speculative since most data available are obtained *in vitro*.

This study has some limitations. First, the number of patients enrolled was small, and the results obtained were interpreted as hypothesis-generating. The incidence of severe HS is relatively low though is increasing due to the climate change. Actually, only about 10 patients with severe HS was admitted into our ICU per year. In the further study, we will consider to expand the sample size. Additionally, only cases at the first HS episode were enrolled in this study to prevent subjects with immunosuppression or chronic inflammation from possibly affecting our results, and hence may limit the generalizability of our results. The plasma exosome counts were not determined either in patients with HS or normal controls; thus, it was impossible to estimate the impacts of HS on exosome number. The contents of exosomal miRNAs and mRNAs were not normalized relative to the plasma exosome amount. Moreover, only mRNAs associated with coagulation activation and inflammatory response were assessed, since they have been suggested by previous studies to regulate exosomes in HS. Using such method, it was feasible to assess additional pathophysiological targets for exosomal mRNAs. To compare the effects between infectious and non-infectious injuries, the control group comprising infected ICU cases was not enrolled in this study. Finally, no functional or mechanistic miRNA studies were taken. We did not perform additional experiments to identify the specific roles of these differentially expressed miRNAs; thus, these markers need to be further explored in the future. Any of these miRNAs may be a potential target in the treatment of HS-induced injury. Time-course and thoroughly step-by-step pathway study can be employed to clarify these issues.

## Conclusions

Exosomes from patients with HS convey genetic material that may be associated with key pathways in the pathogenesis of HS, including inflammatory response, blood coagulation, and platelet activation. Further functional studies are warranted to identify the exact contribution of these vesicles to the exchange of genetic material and intercellular communication during HS.

## Data Availability Statement

The datasets presented in this study can be found in online repositories. The names of the repository/repositories and accession number(s) can be found below: https://www.ncbi.nlm.nih.gov/geo/ (accession number: GSE174224).

## Ethics Statement

All animal experiments were conducted in compliance with the criteria outlined in the Guide for the Care and Use of Laboratory Animals (National Institutes of Health publication 86-23, 1985 revision) and were approved by the Animal Care and Use Committee of the General Hospital of Guangzhou Military Command. Blood sample collection from HS patients was approved by the Ethics Committee of the General Hospital of Guangzhou Military Command. Informed consent was obtained from the patients or their representatives.

## Author Contributions

HT supervised the complete study. YL designed the complete study and performed the NTA, western blotting, statistical analysis, as well as manuscript writing. QW, BL, and HL performed the isolation of exosomes and data collection. XW and HC performed the TEM. LS performed the clinical data and blood sample collection. All authors contributed to the article and approved the submitted version.

## Funding

This study was supported by the National Natural Science Foundation of China (grant No. 81671896), Military Medical Innovation Project (18CXZ032), and Natural Science Foundation of Guangdong Province (2019A1515012088).

## Conflict of Interest

The authors declare that the research was conducted in the absence of any commercial or financial relationships that could be construed as a potential conflict of interest.
