# Supplementary material for: Exosomes Derived From Heat Stroke Cases Carry miRNAs Associated With Inflammation and Coagulation Cascade
Source: Front Immunol. 2021 Jun 22;12:624753. doi: 10.3389/fimmu.2021.624753 (PMC8259592; doi:10.3389/fimmu.2021.624753)
Supplement: Supplementary file 1 [file Table_1.doc]

**Table S1.** miRNAs dysregulated in HS-hepatocyte EVs identified using NGS analysis

| **AccID** | **log2FC** | **P-value** | **FDR** | **Style** |
| --- | --- | --- | --- | --- |
| hsa-miR-511-3p | 10.44555535 | 0.001051524 | 0.014788254 | up |
| hsa-miR-122-5p | 8.276723708 | 0.005868702 | 0.046320826 | up |
| hsa-miR-155-3p | 7.658481446 | 5.18604E-05 | 0.002694095 | up |
| hsa-miR-1290 | 7.535022672 | 1.23381E-07 | 3.42525E-05 | up |
| hsa-let-7b-5p | 7.081014395 | 0.000227207 | 0.004955503 | up |
| hsa-miR-1298-5p | 6.74576804 | 2.58948E-05 | 0.001820878 | up |
| hsa-miR-193a-5p | 6.602101886 | 7.49325E-05 | 0.002851128 | up |
| hsa-miR-855-5p | 6.418510241 | 0.000272081 | 0.005628786 | up |
| hsa-miR-let-7c-5p | 6.297028039 | 2.95983E-09 | 1.52628E-06 | up |
| hsa-miR-363-3p | 6.269409954 | 1.54789E-13 | 1.19729E-10 | up |
| hsa-miR-618 | 6.231316715 | 0.000276527 | 0.005628786 | up |
| hsa-miR-29b-3p | 6.164685475 | 0.000733793 | 0.011519171 | up |
| hsa-miR-99b-3p | 6.147758159 | 4.93893E-14 | 7.64053E-11 | up |
| hsa-miR-485-3p | 6.083130973 | 6.25082E-07 | 0.000123803 | up |
| hsa-miR-1243 | 6.080205186 | 6.40223E-07 | 0.000123803 | up |
| hsa-miR-193a-5p | 6.069448799 | 1.32848E-07 | 3.42525E-05 | up |
| hsa-miR-137-5p | 6.047009206 | 0.000763775 | 0.011815601 | up |
| hsa-miR-590-5p | 6.037288962 | 0.000383296 | 0.007144091 | up |
| hsa-miR-511-5p | 5.905742669 | 1.7233E-06 | 0.000242359 | up |
| hsa-miR-1267 | 5.896199662 | 3.28785E-05 | 0.002088438 | up |
| hsa-miR-148a-3p | 5.865441423 | 0.001261164 | 0.016395129 | up |
| hsa-miR-30d-5p | 5.831116516 | 0.002248497 | 0.024495949 | up |
| hsa-miR-486-5p | 5.564447631 | 1.41289E-05 | 0.001264144 | up |
| hsa-miR-548a-3p | 5.552914827 | 7.19604E-05 | 0.002851128 | up |
| hsa-miR-99a-5p | 5.539603918 | 5.92159E-06 | 0.000654335 | up |
| hsa-miR-520d-5p | 5.53697579 | 0.000148742 | 0.003900067 | up |
| hsa-miR-518b-3p | 5.51551199 | 0.001133096 | 0.015360113 | up |
| hsa-miR-140-3p | 5.481357457 | 0.000181949 | 0.004264777 | up |
| hsa-miR-508-5p | 5.458111243 | 0.006282607 | 0.048114816 | up |
| hsa-miR-592 | 5.447297769 | 0.000167126 | 0.004147149 | up |
| hsa-miR-324-3p | 5.445343033 | 0.002847146 | 0.029760376 | up |
| hsa-miR-296-5p | 5.440772306 | 0.003753563 | 0.036066845 | up |
| hsa-miR-1285-3p | 5.426482304 | 0.001343155 | 0.017066652 | up |
| hsa-miR-598-3p | 5.413461255 | 0.004338065 | 0.03791518 | up |
| hsa-miR-766-3p | 5.384247124 | 0.000518532 | 0.008680444 | up |
| hsa-miR-614 | 5.346661476 | 0.002538486 | 0.027271096 | up |
| hsa-miR-1183 | 5.313071929 | 0.000110185 | 0.003626728 | up |
| hsa-miR-339-3p | 5.306714553 | 0.000124834 | 0.003696458 | up |
| hsa-miR-1233-3p | 5.264430569 | 0.000274062 | 0.005628786 | up |
| hsa-miR-188-3p | 5.226803605 | 0.003360793 | 0.033327859 | up |
| hsa-miR-625-3p | 5.192911687 | 0.000778011 | 0.011916657 | up |
| hsa-miR-875-3p | 5.174336191 | 0.00086226 | 0.012950644 | up |
| hsa-miR-378b | 5.170813667 | 8.04617E-06 | 0.000829828 | up |
| hsa-miR-758-3p | 5.167747909 | 2.39966E-06 | 0.000309356 | up |
| hsa-miR-54a-5p | 5.152096449 | 1.20951E-07 | 3.42525E-05 | up |
| hsa-miR-27a | 5.141395196 | 0.002041626 | 0.023395526 | up |
| hsa-miR-34a | 5.138801488 | 0.00125247 | 0.016395129 | up |
| hsa-miR-125b-5p | 5.122676194 | 1.33884E-05 | 0.001264144 | up |
| hsa-miR-15a | 5.110036409 | 0.000359735 | 0.006786705 | up |
| hsa-miR-558 | 5.051441121 | 0.005306746 | 0.043667749 | up |
| hsa-miR-483-5p | 4.985278141 | 6.82155E-05 | 0.002851128 | up |
| hsa-miR-1277-3p | 4.96612105 | 0.001033352 | 0.014666017 | up |
| hsa-miR-1260a | 4.931265335 | 0.000137917 | 0.003743124 | up |
| hsa-miR-122-3p | 4.882978843 | 6.46948E-05 | 0.002851128 | up |
| hsa-miR-16-5p | 4.870571508 | 4.4788E-05 | 0.002474535 | up |
| hsa-miR-378c | 4.865473522 | 3.50998E-05 | 0.002088438 | up |
| hsa-miR-192-5p | 4.819906165 | 0.000130442 | 0.003696458 | up |
| hsa-miR-122b-5p | 4.813043234 | 7.55632E-05 | 0.002851128 | up |
| hsa-miR-548b-3p | 4.787050472 | 0.005772356 | 0.046320826 | up |
| hsa-miR-194-5p | 4.781402495 | 0.00015155 | 0.003907475 | up |
| hsa-miR-658 | 4.766242612 | 0.004135298 | 0.036983224 | up |
| hsa-miR-195-5p | 4.723631785 | 0.003657473 | 0.035363195 | up |
| hsa-miR-19a-3p | 4.701557418 | 0.003604788 | 0.035294977 | up |
| hsa-miR-885-5p | 4.670729991 | 6.79172E-05 | 0.002851128 | up |
| hsa-miR-25-3p | 4.668210424 | 0.002787529 | 0.029335427 | up |
| hsa-miR-676-3p | 4.635212949 | 0.000147017 | 0.003900067 | up |
| hsa-miR-30a-5p | 4.633464441 | 0.000104981 | 0.003530551 | up |
| hsa-miR-378e | 4.573833884 | 6.24059E-05 | 0.002851128 | up |
| hsa-miR-320a | 4.545202766 | 7.5698E-07 | 0.000130116 | up |
| hsa-miR-518d-3p | 4.523946854 | 1.83062E-05 | 0.001415983 | up |
| hsa-miR-532-3p | 4.438273684 | 0.005937185 | 0.046435358 | up |
| hsa-miR-152-3p | 4.434831855 | 1.47089E-05 | 0.001264144 | up |
| hsa-miR-885-3p | 4.343597305 | 0.000104869 | 0.003530551 | up |
| hsa-miR-548c-3p | 4.298413102 | 0.001422001 | 0.017598688 | up |
| hsa-miR-597-5p | 4.291099833 | 0.00423662 | 0.037238931 | up |
| hsa-miR-636 | 4.286856265 | 0.00041197 | 0.00741067 | up |
| hsa-miR-645 | 4.261328603 | 0.000119754 | 0.003696458 | up |
| hsa-miR-720 | 4.245115 | 0.004098041 | 0.036983224 | up |
| hsa-miR-378g | 4.242244907 | 7.1321E-05 | 0.002851128 | up |
| hsa-miR-449b-3p | 4.190458798 | 0.005779387 | 0.046320826 | up |
| hsa-miR-886-5p | 4.181509179 | 0.000737167 | 0.011519171 | up |
| hsa-miR-133a-5p | 4.163298014 | 5.89909E-05 | 0.002851128 | up |
| hsa-miR-92a-3p | 4.117957927 | 0.001603042 | 0.019526823 | up |
| hsa-miR-549a | 4.093083013 | 0.002382692 | 0.025776395 | up |
| hsa-miR-455-3p | 4.085035465 | 0.000201408 | 0.004650421 | up |
| hsa-miR-875-5p | 4.084769606 | 0.004764889 | 0.04028024 | up |
| hsa-miR-378j | 4.067935221 | 0.000172496 | 0.004147149 | up |
| hsa-miR-100-5p | 4.064359746 | 0.000528185 | 0.008692578 | up |
| hsa-miR-378f | 4.058976779 | 0.000214544 | 0.004880873 | up |
| hsa-miR-323b-5p | 4.049295395 | 0.002076178 | 0.023569945 | up |
| hsa-miR-378d | 3.967150304 | 0.000303754 | 0.006024464 | up |
| hsa-miR-378a-3p | 3.887692985 | 0.000172964 | 0.004147149 | up |
| hsa-miR-99b-5p | 3.87989185 | 0.000860374 | 0.012950644 | up |
| hsa-miR-675-3p | 3.863440724 | 0.000984691 | 0.014370908 | up |
| hsa-miR-125b-1-3p | 3.858649521 | 0.000355423 | 0.006786705 | up |
| hsa-miR-133a-3p | 3.856130758 | 0.000397748 | 0.007316155 | up |
| hsa-miR-156-3p | 3.828665539 | 0.001070782 | 0.014909039 | up |
| hsa-miR-422a | 3.821697972 | 0.000227434 | 0.004955503 | up |
| hsa-miR-508-5p | 3.773055535 | 0.004143019 | 0.036983224 | up |
| hsa-miR-455-5p | 3.74855908 | 3.54643E-06 | 0.000422025 | up |
| hsa-miR-30a-5p | 3.722860052 | 1.74445E-05 | 0.001415983 | up |
| hsa-miR-362-3p | 3.708667691 | 0.002089088 | 0.023569945 | up |
| hsa-miR-210-3p | 3.706738617 | 0.001018893 | 0.014594692 | up |
| hsa-miR-80 | 3.690830266 | 0.004398526 | 0.038227641 | up |
| hsa-miR-428 | 3.690701315 | 0.00017425 | 0.004147149 | up |
| hsa-miR-686-5p | 3.563674461 | 0.004159716 | 0.036983224 | up |
| hsa-miR-557-3p | 3.54859907 | 0.005170255 | 0.042854747 | up |
| hsa-miR-127-5p | 3.534359038 | 0.005180244 | 0.042854747 | up |
| hsa-miR-574-5p | 3.460709408 | 0.001686106 | 0.020064661 | up |
| hsa-miR-95-5p | 3.450983644 | 0.004151843 | 0.036983224 | up |
| hsa-miR-672-5p | 3.426905205 | 0.000131419 | 0.003696458 | up |
| hsa-miR-378h | 3.399669168 | 0.001305573 | 0.016831014 | up |
| hsa-miR-499b-3p | 3.367588817 | 0.000512354 | 0.008680444 | up |
| hsa-miR-499a-5p | 3.321524527 | 0.000401987 | 0.007316155 | up |
| hsa-miR-873-5p | 3.303504919 | 0.002108672 | 0.023569945 | up |
| hsa-miR-468 | 3.242848143 | 0.000313963 | 0.006071253 | up |
| hsa-miR-188-5p | 3.202653388 | 0.001920073 | 0.022166816 | up |
| hsa-miR-501-5p | 3.201586587 | 0.001662516 | 0.019937302 | up |
| hsa-miR-375-3p | 3.198625055 | 0.004051092 | 0.036983224 | up |
| hsa-miR-601 | 3.183032738 | 0.001142479 | 0.015360113 | up |
| hsa-miR-313-3p | 3.181596542 | 0.003044582 | 0.031191848 | up |
| hsa-miR-125b-5p | 3.177573994 | 0.001729595 | 0.020425067 | up |
| hsa-miR-480-3p | 3.139771854 | 0.000225445 | 0.004955503 | up |
| hsa-miR-141-5p | 3.135945816 | 0.004088257 | 0.036983224 | up |
| hsa-miR-475-5p | 3.123371628 | 0.004584576 | 0.039401888 | up |
| hsa-miR-660-3p | 3.107484568 | 0.001847427 | 0.021651279 | up |
| hsa-miR-208b-3p | 3.076327192 | 0.003236933 | 0.032306675 | up |
| hsa-miR-619-5p | 3.068163387 | 0.000131118 | 0.003696458 | up |
| hsa-miR-36a-5p | 3.0546317 | 0.000115998 | 0.003666773 | up |
| hsa-miR-650-3p | 3.050923963 | 0.003925388 | 0.036983224 | up |
| hsa-miR-497-5p | 3.01762823 | 2.81112E-05 | 0.001890783 | up |
| hsa-miR-480-5p | 3.014405265 | 0.001079387 | 0.014909039 | up |
| hsa-miR-148a-3p | 2.988162684 | 0.00594996 | 0.046435358 | up |
| hsa-miR-124-5p | 2.951062151 | 0.00395291 | 0.036983224 | up |
| hsa-miR-21-3p | 2.934507665 | 0.004686581 | 0.039835941 | up |
| hsa-miR-391 | 2.929568628 | 0.000559096 | 0.009009607 | up |
| hsa-miR-193b-3p | 2.918888063 | 0.005446315 | 0.044344472 | up |
| hsa-miR-95-3p | 2.908344044 | 0.000872945 | 0.01298506 | up |
| hsa-miR-365b-3p | 2.888195604 | 0.001576713 | 0.019358531 | up |
| hsa-miR-675-5p | 2.883829012 | 0.001414023 | 0.017598688 | up |
| hsa-miR-500a-3p | 2.828996605 | 0.005840891 | 0.046320826 | up |
| hsa-miR-463 | 2.820851921 | 8.29863E-05 | 0.002989247 | up |
| hsa-miR-770 | 2.80885692 | 0.000460923 | 0.008011765 | up |
| hsa-miR-133b | 2.733475223 | 0.002157145 | 0.023667401 | up |
| hsa-miR-467 | 2.727764422 | 0.006033295 | 0.046435358 | up |
| hsa-miR-130-5p | 2.583022076 | 0.004642189 | 0.039676606 | up |
| hsa-miR-125a-5p | 2.581138745 | 0.003159096 | 0.031941968 | up |
| hsa-miR-99 | 2.579135083 | 0.003921596 | 0.036983224 | up |
| hsa-miR-365a-3p | 2.507688657 | 0.005501005 | 0.044555261 | up |
| hsa-miR-448 | 2.39972093 | 0.004084038 | 0.036983224 | up |
| hsa-miR-394-5p | 2.283892989 | 0.006021612 | 0.046435358 | up |
| hsa-miR-29a-3p | 2.268261556 | 0.00115176 | 0.015360113 | up |
| hsa-miR-512-3p | 2.126801502 | 0.005430677 | 0.044344472 | up |
| hsa-miR-475-3p | 1.990553632 | 0.005843288 | 0.046320826 | up |
| hsa-miR-30e-3p | -1.835550942 | 0.004945721 | 0.0415597 | down |
| hsa-miR-222-3p | -1.856156913 | 0.00164635 | 0.019897684 | down |
| hsa-miR-584-5p | -2.009084984 | 0.004226844 | 0.037238931 | down |
| hsa-miR-671-3p | -2.014744985 | 0.002636901 | 0.02813301 | down |
| hsa-miR-340-5p | -2.030456926 | 0.003124884 | 0.031803918 | down |
| hsa-miR-654-5p | -2.110003521 | 0.003565424 | 0.035131917 | down |
| hsa-miR-30b-3p | -2.192120837 | 0.004969971 | 0.0415597 | down |
| hsa-miR-766-5p | -2.212703259 | 0.000521837 | 0.008680444 | down |
| hsa-miR-30c-1-3p | -2.31408293 | 0.003994987 | 0.036983224 | down |
| hsa-miR-374b-5p | -2.343572476 | 0.003917189 | 0.036983224 | down |
| hsa-miR-432-5p | -2.358199166 | 0.002717244 | 0.028791614 | down |
| hsa-miR-374c-3p | -2.358739972 | 0.002871652 | 0.029815068 | down |
| hsa-miR-331-3p | -2.435047906 | 0.001003414 | 0.014507302 | down |
| hsa-miR-130b-5p | -2.469418509 | 0.002127476 | 0.023569945 | down |
| hsa-miR-224-3p | -2.477360429 | 0.006010819 | 0.046435358 | down |
| hsa-miR-135a-5p | -2.506163407 | 0.001895315 | 0.022045503 | down |
| hsa-miR-199a-5p | -2.513372321 | 0.003633528 | 0.035352625 | down |
| hsa-miR-98-5p | -2.557471672 | 0.000449668 | 0.007904952 | down |
| hsa-miR-186-5p | -2.72167449 | 0.001115982 | 0.015278093 | down |
| hsa-miR-487b-3p | -2.729215601 | 0.000241089 | 0.005180058 | down |
| hsa-miR-454-3p | -2.748583439 | 0.002930923 | 0.030227591 | down |
| hsa-miR-495-3p | -2.758027548 | 0.000420334 | 0.00747421 | down |
| hsa-miR-26a-1-3p | -2.763384301 | 0.003216004 | 0.03230622 | down |
| hsa-miR-409-3p | -2.820796229 | 0.001345916 | 0.017066652 | down |
| hsa-miR-543 | -2.850172237 | 0.000680847 | 0.010858462 | down |
| hsa-miR-296-5p | -2.931642939 | 8.30883E-05 | 0.002989247 | down |
| hsa-miR-1185-1-3p | -2.947501718 | 0.000538761 | 0.008773296 | down |
| hsa-miR-329-3p | -3.010672012 | 0.000102978 | 0.003530551 | down |
| hsa-miR-548j-5p | -3.066580896 | 0.001162522 | 0.015371118 | down |
| hsa-miR-556-5p | -3.086513534 | 0.00095938 | 0.014134865 | down |
| hsa-miR-654-3p | -3.09343416 | 2.53667E-05 | 0.001820878 | down |
| hsa-miR-340-3p | -3.09443468 | 0.000302301 | 0.006024464 | down |
| hsa-miR-200a-5p | -3.107804542 | 6.20481E-05 | 0.002851128 | down |
| hsa-miR-221-3p | -3.134305694 | 0.001390292 | 0.017486025 | down |
| hsa-miR-26a-5p | -3.171505823 | 4.01985E-05 | 0.002303226 | down |
| hsa-miR-758-3p | -3.216623778 | 0.000256334 | 0.005432171 | down |
| hsa-miR-323b-3p | -3.255988987 | 0.000508585 | 0.008680444 | down |
| hsa-miR-224-5p | -3.363071212 | 0.002133027 | 0.023569945 | down |
| hsa-miR-485-3p | -3.422140644 | 0.004446777 | 0.038431083 | down |
| hsa-miR-487a-5p | -3.426603802 | 0.000123266 | 0.003696458 | down |
| hsa-miR-194-5p | -3.902033656 | 0.000116142 | 0.003666773 | down |
| hsa-miR-411-3p | -3.936102911 | 5.22449E-05 | 0.002694095 | down |
| hsa-miR-196a-5p | -3.960486658 | 7.3528E-05 | 0.002851128 | down |
| hsa-miR-379-3p | -4.028025819 | 3.48353E-05 | 0.002088438 | down |
| hsa-miR-16-5p | -4.037789955 | 0.000311387 | 0.006071253 | down |
| hsa-miR-151a-3p | -4.170054878 | 1.18001E-06 | 0.000182547 | down |
| hsa-miR-146a-5p | -4.181358731 | 0.000158479 | 0.004019119 | down |
| hsa-miR-150-3p | -4.335973238 | 0.000134263 | 0.003709015 | down |

**Table S2.** Differentially expressed targeted mRNAs associated with inflammatory response

| **Gene symbol** | **Protein Name** |
| --- | --- |
| NFAM1 | NFAT activating protein with ITAM motif 1 |
| LYN | LYN proto-oncogene, Src family tyrosine kinase |
| NRROS | negative regulator of reactive oxygen species |
| DAB2IP | DAB2 interacting protein |
| IGFBP4 | insulin-like growth factor binding protein 4 |
| PRKD1 | protein kinase D1 |
| SP100 | SP100 nuclear antigen |
| IL25 | interleukin 25 |
| CRLF2 | cytokine receptor-like factor 2 |
| PSTPIP1 | proline-serine-threonine phosphatase interacting protein 1 |
| NLRP2 | NLR family, pyrin domain containing 2 |
| NFKBIZ | nuclear factor of kappa light polypeptide gene enhancer in B-cells inhibitor, zeta |
| AOAH | acyloxyacyl hydrolase (neutrophil) |
| PIK3C2A | phosphatidylinositol-4-phosphate 3-kinase, catalytic subunit type 2 alpha |
| CXCR1 | chemokine (C-X-C motif) receptor 1 |
| THEMIS2 | thymocyte selection associated family member 2 |
| RELA | v-rel avian reticuloendotheliosis viral oncogene homolog A |
| IL17C | interleukin 17C |
| CCR4 | chemokine (C-C motif) receptor 4 |
| NDST1 | N-deacetylase/N-sulfotransferase (heparan glucosaminyl) 1 |
| FOLR2 | folate receptor 2 (fetal) |
| CHST4 | carbohydrate (N-acetylglucosamine 6-O) sulfotransferase 4 |
| TBXA2R | thromboxane A2 receptor |
| CCL5 | chemokine (C-C motif) ligand 5 |
| APOL3 | apolipoprotein L, 3 |
| IL13 | interleukin 13 |
| TNIP2 | TNFAIP3 interacting protein 2 |
| TGFB1 | transforming growth factor, beta 1 |
| HRH1 | histamine receptor H1 |
| CCL21 | chemokine (C-C motif) ligand 21 |
| PROK2 | prokineticin 2 |
| CXCR6 | chemokine (C-X-C motif) receptor 6 |
| BMPR1B | bone morphogenetic protein receptor, type IB |
| PTGDR | prostaglandin D2 receptor (DP) |
| PRDX5 | peroxiredoxin 5 |
| CXCL6 | chemokine (C-X-C motif) ligand 6 |
| CXCL10 | chemokine (C-X-C motif) ligand 10 |
| ADORA2A | adenosine A2a receptor |
| NLRP4 | NLR family, pyrin domain containing 4 |
| CCL2 | chemokine (C-C motif) ligand 2 |
| CYBA | cytochrome b-245, alpha polypeptide |
| JAK3 | Janus kinase 3 |
| PIK3CD | phosphatidylinositol-4,5-bisphosphate 3-kinase, catalytic subunit delta |
| CXCR4 | chemokine (C-X-C motif) receptor 4 |
| CCR5 | chemokine (C-C motif) receptor 5 (gene/pseudogene) |
| TNFAIP6 | tumor necrosis factor, alpha-induced protein 6 |
| APOC3 | apolipoprotein C-III |
| IKBKG | inhibitor of kappa light polypeptide gene enhancer in B-cells, kinase gamma |
| CD40LG | CD40 ligand |
| CHST2 | carbohydrate (N-acetylglucosamine-6-O) sulfotransferase 2 |
| AIMP1 | aminoacyl tRNA synthetase complex-interacting multifunctional protein 1 |
| ADORA1 | adenosine A1 receptor |
| TSPAN2 | tetraspanin 2 |
| TLR7 | Toll-like receptor 7 |
| CXCL11 | chemokine (C-X-C motif) ligand 11 |
| KIT | v-kit Hardy-Zuckerman 4 feline sarcoma viral oncogene homolog |
| KDM6B | lysine (K)-specific demethylase 6B |
| LGALS9 | lectin, galactoside-binding, soluble, 9 |
| AFAP1L2 | actin filament associated protein 1-like 2 |
| VNN1 | vanin 1 |
| S100A9 | S100 calcium binding protein A9 |
| PIK3CB | phosphatidylinositol-4,5-bisphosphate 3-kinase, catalytic subunit beta |
| NOD1 | nucleotide binding oligomerization domain containing 1 |
| TNIP3 | TNFAIP3 interacting protein 3 |
| CCL26 | chemokine (C-C motif) ligand 26 |
| NFKB2 | nuclear factor of kappa light polypeptide gene enhancer in B-cells 2 (p49/p100) |
| NFATC3 | nuclear factor of activated T cells, cytoplasmic, calcineurin-dependent 3 |
| FFAR2 | free fatty acid receptor 2 |
| IL1RAP | interleukin 1 receptor accessory protein |
| IL2RA | interleukin 2 receptor, alpha |
| CCL22 | chemokine (C-C motif) ligand 22 |
| PIK3C2G | phosphatidylinositol-4-phosphate 3-kinase, catalytic subunit type 2 gamma |
| TLR5 | toll-like receptor 5 |
| TPST1 | tyrosylprotein sulfotransferase 1 |
| BMP6 | bone morphogenetic protein 6 |
| CCR3 | chemokine (C-C motif) receptor 3 |
| HDAC9 | histone deacetylase 9 |
| CCL7 | chemokine (C-C motif) ligand 7 |
| OLR1 | oxidized low density lipoprotein (lectin-like) receptor 1 |
| IDO1 | indoleamine 2,3-dioxygenase 1 |
| IL23R | interleukin 23 receptor |
| TRIL | TLR4 interactor with leucine-rich repeats |
| TUSC2 | tumor suppressor candidate 2 |
| C5AR1 | complement component 5a receptor 1 |
| S1PR3 | sphingosine-1-phosphate receptor 3 |
| IL10RB | interleukin 10 receptor, beta |
| ELF3 | E74-like factor 3 (ets domain transcription factor, epithelial-specific ) |
| JMJD7-PLA2G4B | JMJD7-PLA2G4B readthrough |
| CD40 | CD40 molecule, TNF receptor superfamily member 5 |
| SCN9A | sodium channel, voltage-gated, type IX, alpha subunit |
| ITGAL | integrin, alpha L (antigen CD11A (p180), lymphocyte function-associated antigen 1; alpha polypeptide) |
| CAMK1D | calcium/calmodulin-dependent protein kinase ID |
| LY86 | lymphocyte antigen 86 |
| PLA2G4C | phospholipase A2, group IVC (cytosolic, calcium-independent) |
| TNFRSF1A | tumor necrosis factor receptor superfamily, member 1A |
| PIK3CA | phosphatidylinositol-4,5-bisphosphate 3-kinase, catalytic subunit alpha |
| RPS6KA5 | ribosomal protein S6 kinase, 90 kDa, polypeptide 5 |
| FPR2 | formyl peptide receptor 2 |
| TNFRSF1B | tumor necrosis factor receptor superfamily, member 1B |
| GPR68 | G protein-coupled receptor 68 |
| ATRN | attractin |
| SYK | spleen tyrosine kinase |
| XCL2 | chemokine (C motif) ligand 2 |
| MS4A2 | membrane-spanning 4-domains, subfamily A, member 2 |
| F2RL1 | coagulation factor II (thrombin) receptor-like 1 |
| IL36A | interleukin 36, alpha |
| PXK | PX domain containing serine/threonine kinase |
| HDAC5 | histone deacetylase 5 |
| PRKCQ | protein kinase C, theta |
| SPHK1 | sphingosine kinase 1 |
| ADAM8 | ADAM metallopeptidase domain 8 |
| CXCL9 | chemokine (C-X-C motif) ligand 9 |
| TICAM2 | toll-like receptor adaptor molecule 2 |
| PLA2G2D | phospholipase A2, group IID |
| CCL25 | chemokine (C-C motif) ligand 25 |
| IL22 | interleukin 22 |
| MIF | macrophage migration inhibitory factor (glycosylation-inhibiting factor) |
| LYZ | lysozyme |
| NGF | nerve growth factor (beta polypeptide) |
| PDPN | podoplanin |
| PRKCZ | protein kinase C, zeta |
| PIK3C2B | phosphatidylinositol-4-phosphate 3-kinase, catalytic subunit type 2 beta |
| KNG1 | kininogen 1 |
| CCL3L3 | chemokine (C-C motif) ligand 3-like 3 |
| NFATC4 | nuclear factor of activated T cells, cytoplasmic, calcineurin-dependent 4 |
| AZU1 | azurocidin 1 |
| IL5 | interleukin 5 |
| ADGRE2 | EGF-like module containing, mucin-like, hormone receptor-like 2 |
| CEBPB | CCAAT/enhancer binding protein (C/EBP), beta |
| HRH4 | histamine receptor H4 |
| CCR1 | chemokine (C-C motif) receptor 1 |
| CSF1 | colony stimulating factor 1 (macrophage) |
| IL10 | interleukin 10 |
| THBS1 | thrombospondin 1 |
| NAIP | NLR family, apoptosis inhibitory protein |
| HYAL3 | hyaluronoglucosaminidase 3 |
| CALCA | calcitonin-related polypeptide alpha |
| MMP17 | matrix metallopeptidase 17 (membrane-inserted) |
| ALOX15 | arachidonate 15-lipoxygenase |
| IL24 | interleukin 24 |
| CNR2 | cannabinoid receptor 2 (macrophage) |
| CYP26B1 | cytochrome P450, family 26, subfamily B, polypeptide 1 |
| CCL24 | chemokine (C-C motif) ligand 24 |
| IL18 | interleukin 18 |
| TNFRSF4 | tumor necrosis factor receptor superfamily, member 4 |
| BCL6 | B cell CLL/lymphoma 6 |
| CCL4L2 | chemokine (C-C motif) ligand 4-like 2 |
| ITGB2 | integrin, beta 2 (complement component 3 receptor 3 and 4 subunit) |
| IL20 | interleukin 20 |
| HCK | HCK proto-oncogene, Src family tyrosine kinase |
| IL23A | interleukin 23, alpha subunit p19 |
| GGT5 | gamma-glutamyltransferase 5 |
| BDKRB2 | bradykinin receptor B2 |
| TLR9 | toll-like receptor 9 |
| GPER1 | G protein-coupled estrogen receptor 1 |
| CCL8 | chemokine (C-C motif) ligand 8 |
| TLR8 | toll-like receptor 8 |
| TLR1 | toll-like receptor 1 |
| NFKBID | nuclear factor of kappa light polypeptide gene enhancer in B-cells inhibitor, delta |
| MEFV | Mediterranean fever |
| HDAC4 | histone deacetylase 4 |
| IL18RAP | interleukin 18 receptor accessory protein |
| CSF1R | colony stimulating factor 1 receptor |
| KLRG1 | killer cell lectin-like receptor subfamily G, member 1 |
| NOX4 | NADPH oxidase 4 |
| C3AR1 | complement component 3a receptor 1 |
| ADGRE5 | CD97 molecule |
| TLR10 | toll-like receptor 10 |
| KCNJ10 | potassium inwardly-rectifying channel, subfamily J, member 10 |
| ITGB6 | integrin, beta 6 |
| C4A | complement component 4A (Rodgers blood group) |
| CXCR2 | chemokine (C-X-C motif) receptor 2 |
| P2RX7 | purinergic receptor P2X, ligand-gated ion channel, 7 |
| MMP25 | matrix metallopeptidase 25 |
| CXCL5 | chemokine (C-X-C motif) ligand 5 |
| SELE | selectin E |
| NCF1 | neutrophil cytosolic factor 1 |
| SDC1 | syndecan 1 |
| ALOX5 | arachidonate 5-lipoxygenase |
| BLNK | B cell linker |
| IL17D | interleukin 17D |
| RPS6KA4 | ribosomal protein S6 kinase, 90kDa, polypeptide 4 |
| IL1RL2 | interleukin 1 receptor-like 2 |
| F2R | coagulation factor II (thrombin) receptor |
| CXCR3 | chemokine (C-X-C motif) receptor 3 |
| FFAR3 | free fatty acid receptor 3 |
| SCG2 | secretogranin II |
| PTAFR | platelet-activating factor receptor |
| SCUBE1 | signal peptide, CUB domain, EGF-like 1 |
| TICAM1 | toll-like receptor adaptor molecule 1 |
| IL17A | interleukin 17A |
| NLRP3 | NLR family, pyrin domain containing 3 |
| AKT1 | v-akt murine thymoma viral oncogene homolog 1 |
| NFE2L1 | nuclear factor, erythroid 2-like 1 |
| TLR6 | toll-like receptor 6 |
| SERPINA3 | serpin peptidase inhibitor, clade A (alpha-1 antiproteinase, antitrypsin), member 3 |
| TNFAIP3 | tumor necrosis factor, alpha-induced protein 3 |
| PLA2G4B | phospholipase A2, group IVB (cytosolic) |
| PTGS1 | prostaglandin-endoperoxide synthase 1 (prostaglandin G/H synthase and cyclooxygenase) |
| TLR2 | toll-like receptor 2 |
| MECOM | MDS1 and EVI1 complex locus |
| REL | v-rel avian reticuloendotheliosis viral oncogene homolog |
| CCL16 | chemokine (C-C motif) ligand 16 |
| MGLL | monoglyceride lipase |
| CYBB | cytochrome b-245, beta polypeptide |
| XCR1 | chemokine (C motif) receptor 1 |
| CAMK4 | calcium/calmodulin-dependent protein kinase IV |
| CHST1 | carbohydrate (keratan sulfate Gal-6) sulfotransferase 1 |
| IL6 | interleukin 6 |
| IFI16 | interferon, gamma-inducible protein 16 |
| NCR3 | natural cytotoxicity triggering receptor 3 |
| TP73 | tumor protein p73 |
| TACR1 | tachykinin receptor 1 |
| CCL13 | chemokine (C-C motif) ligand 13 |
| C4B | complement component 4B (Chido blood group) |
| NFRKB | nuclear factor related to kappaB binding protein |
| CHIA | chitinase, acidic |
| LAT | linker for activation of T cells |
| CCR2 | chemokine (C-C motif) receptor 2 |
| F11R | F11 receptor |
| LTB4R | leukotriene B4 receptor |
| MEP1B | meprin A, beta |
| CCL19 | chemokine (C-C motif) ligand 19 |
| TNFSF4 | tumor necrosis factor (ligand) superfamily, member 4 |
| CRP | C-reactive protein, pentraxin-related |
| RARRES2 | retinoic acid receptor responder (tazarotene induced) 2 |
| PIK3CG | phosphatidylinositol-4,5-bisphosphate 3-kinase, catalytic subunit gamma |
| LY75 | lymphocyte antigen 75 |
| SEMA7A | semaphorin 7A, GPI membrane anchor (John Milton Hagen blood group) |
| HNRNPA0 | heterogeneous nuclear ribonucleoprotein A0 |
| RAC1 | ras-related C3 botulinum toxin substrate 1 (rho family, small GTP binding protein Rac1) |
| SIGLEC1 | sialic acid binding Ig-like lectin 1, sialoadhesin |
| TNIP1 | TNFAIP3 interacting protein 1 |
| NLRP1 | NLR family, pyrin domain containing 1 |
| SELP | selectin P (granule membrane protein 140kDa, antigen CD62) |
| TLR3 | toll-like receptor 3 |
| CCR7 | chemokine (C-C motif) receptor 7 |
| SERPINA1 | serpin peptidase inhibitor, clade A (alpha-1 antiproteinase, antitrypsin), member 1 |
| PTGS2 | prostaglandin-endoperoxide synthase 2 (prostaglandin G/H synthase and cyclooxygenase) |
| ACKR2 | atypical chemokine receptor 2 |
| ITCH | itchy E3 ubiquitin protein ligase |
| AOX1 | aldehyde oxidase 1 |
| IL1B | interleukin 1, beta |
| RXRA | retinoid X receptor, alpha |
| MYD88 | myeloid differentiation primary response 88 |
| CCL17 | chemokine (C-C motif) ligand 17 |
| SLC11A1 | solute carrier family 11 (proton-coupled divalent metal ion transporter), member 1 |
| NTRK2 | neurotrophic tyrosine kinase, receptor, type 2 |
| LIPA | lipase A, lysosomal acid, cholesterol esterase |
| CYP4F11 | cytochrome P450, family 4, subfamily F, polypeptide 11 |
| CD180 | CD180 molecule |
| HYAL1 | hyaluronoglucosaminidase 1 |
| IL34 | interleukin 34 |
| KLKB1 | kallikrein B, plasma (Fletcher factor) 1 |
| ADORA3 | adenosine A3 receptor |
| IL27 | interleukin 27 |
| IL1A | interleukin 1, alpha |
| PLP1 | proteolipid protein 1 |
| REG3A | regenerating islet-derived 3 alpha |
| RELB | v-rel avian reticuloendotheliosis viral oncogene homolog B |
| MAP2K3 | mitogen-activated protein kinase kinase 3 |
| CHUK | conserved helix-loop-helix ubiquitous kinase |
| CRH | corticotropin releasing hormone |
| HMGB1 | high mobility group box 1 |
| BMP2 | bone morphogenetic protein 2 |
| IL19 | interleukin 19 |
| IRAK2 | interleukin-1 receptor-associated kinase 2 |
| CCL18 | chemokine (C-C motif) ligand 18 (pulmonary and activation-regulated) |
| NOS2 | nitric oxide synthase 2, inducible |
| CHI3L1 | chitinase 3-like 1 (cartilage glycoprotein-39) |
| LY75-CD302 | LY75-CD302 readthrough |
| AXL | AXL receptor tyrosine kinase |
| MAPKAPK2 | mitogen-activated protein kinase-activated protein kinase 2 |

**Table S3.** Differentially expressed targeted mRNAs associated with platelet activation

| **LYN** | **LYN proto-oncogene, Src family tyrosine kinase** |  |
| --- | --- | --- |
| MMRN1 | multimerin 1 |  |
| VWF | von Willebrand factor |  |
| RAF1 | Raf-1 proto-oncogene, serine/threonine kinase |  |
| ENTPD1 | ectonucleoside triphosphate diphosphohydrolase 1 |  |
| RASGRP1 | RAS guanyl releasing protein 1 (calcium and DAG-regulated) |  |
| PFN1 | profilin 1 |  |
| EGF | epidermal growth factor |  |
| PIK3C2A | phosphatidylinositol-4-phosphate 3-kinase, catalytic subunit type 2 alpha |  |
| DGKH | diacylglycerol kinase, eta |  |
| MAPK14 | mitogen-activated protein kinase 14 |  |
| GNAI3 | guanine nucleotide binding protein (G protein), alpha inhibiting activity polypeptide 3 |  |
| SERPING1 | serpin peptidase inhibitor, clade G (C1 inhibitor), member 1 |  |
| TBXA2R | thromboxane A2 receptor |  |
| IGF2 | insulin-like growth factor 2 |  |
| PRKCD | protein kinase C, delta |  |
| RAPGEF4 | Rap guanine nucleotide exchange factor (GEF) 4 |  |
| TGFB1 | transforming growth factor, beta 1 |  |
| GP1BB | glycoprotein Ib (platelet), beta polypeptide |  |
| SPARC | secreted protein, acidic, cysteine-rich (osteonectin) |  |
| F5 | coagulation factor V (proaccelerin, labile factor) |  |
| P2RY12 | purinergic receptor P2Y, G protein coupled, 12 |  |
| MAPK1 | mitogen-activated protein kinase 1 |  |
| RAP2B | RAP2B, member of RAS oncogene family |  |
| APBB1IP | amyloid beta (A4) precursor protein-binding, family B, member 1 interacting protein |  |
| PIK3CD | phosphatidylinositol-4,5-bisphosphate 3-kinase, catalytic subunit delta |  |
| PPIA | peptidylprolyl isomerase A (cyclophilin A) |  |
| YWHAZ | tyrosine 3-monooxygenase/tryptophan 5-monooxygenase activation protein, zeta |  |
| ENTPD2 | ectonucleoside triphosphate diphosphohydrolase 2 |  |
| CD9 | CD9 molecule |  |
| GNA11 | guanine nucleotide binding protein (G protein), alpha 11 (Gq class) |  |
| CSK | c-src tyrosine kinase |  |
| CD40LG | CD40 ligand |  |
| CFL1 | cofilin 1 (non-muscle) |  |
| ARRB2 | arrestin, beta 2 |  |
| PTK2 | protein tyrosine kinase 2 |  |
| BCAR1 | breast cancer anti-estrogen resistance 1 |  |
| RASGRP2 | RAS guanyl releasing protein 2 (calcium and DAG-regulated) |  |
| PIK3R5 | phosphoinositide-3-kinase, regulatory subunit 5 |  |
| PIK3CB | phosphatidylinositol-4,5-bisphosphate 3-kinase, catalytic subunit beta |  |
| LAMP2 | lysosomal-associated membrane protein 2 |  |
| DAGLA | diacylglycerol lipase, alpha |  |
| HGF | hepatocyte growth factor (hepapoietin A; scatter factor) |  |
| ACTN2 | actinin, alpha 2 |  |
| CRK | v-crk avian sarcoma virus CT10 oncogene homolog |  |
| PIK3C2G | phosphatidylinositol-4-phosphate 3-kinase, catalytic subunit type 2 gamma |  |
| DGKI | diacylglycerol kinase, iota |  |
| TLN1 | talin 1 |  |
| F2RL2 | coagulation factor II (thrombin) receptor-like 2 |  |
| PLSCR1 | phospholipid scramblase 1 |  |
| TGFB3 | transforming growth factor, beta 3 |  |
| RHOG | ras homolog family member G |  |
| TGFB2 | transforming growth factor, beta 2 |  |
| PIK3R6 | phosphoinositide-3-kinase, regulatory subunit 6 |  |
| TRPC3 | transient receptor potential cation channel, subfamily C, member 3 |  |
| DGKA | diacylglycerol kinase, alpha 80kDa |  |
| GNG2 | guanine nucleotide binding protein (G protein), gamma 2 |  |
| CD40 | CD40 molecule, TNF receptor superfamily member 5 |  |
| ACTN1 | actinin, alpha 1 |  |
| DGKK | diacylglycerol kinase, kappa |  |
| PIK3CA | phosphatidylinositol-4,5-bisphosphate 3-kinase, catalytic subunit alpha |  |
| ITPR3 | inositol 1,4,5-trisphosphate receptor, type 3 |  |
| SYK | spleen tyrosine kinase |  |
| PTPN1 | protein tyrosine phosphatase, non-receptor type 1 |  |
| SOS1 | son of sevenless homolog 1 (Drosophila) |  |
| PDPK1 | 3-phosphoinositide dependent protein kinase 1 |  |
| CALM2 | calmodulin 2 (phosphorylase kinase, delta) |  |
| PRKCQ | protein kinase C, theta |  |
| RHOB | ras homolog family member B |  |
| SERPINF2 | serpin peptidase inhibitor, clade F (alpha-2 antiplasmin, pigment epithelium derived factor), member 2 |  |
| COL1A1 | collagen, type I, alpha 1 |  |
| PRKCZ | protein kinase C, zeta |  |
| PIK3C2B | phosphatidylinositol-4-phosphate 3-kinase, catalytic subunit type 2 beta |  |
| LEFTY2 | left-right determination factor 2 |  |
| VAV3 | vav 3 guanine nucleotide exchange factor |  |
| KNG1 | kininogen 1 |  |
| DGKG | diacylglycerol kinase, gamma 90kDa |  |
| RHOA | ras homolog family member A |  |
| FCER1G | Fc fragment of IgE, high affinity I, receptor for; gamma polypeptide |  |
| ADRA2A | adrenoceptor alpha 2A |  |
| ITPR1 | inositol 1,4,5-trisphosphate receptor, type 1 |  |
| THBS1 | thrombospondin 1 |  |
| GNAQ | guanine nucleotide binding protein (G protein), q polypeptide |  |
| TIMP1 | TIMP metallopeptidase inhibitor 1 |  |
| VAV1 | vav 1 guanine nucleotide exchange factor |  |
| GNA14 | guanine nucleotide binding protein (G protein), alpha 14 |  |
| GNAT3 | guanine nucleotide binding protein, alpha transducing 3 |  |
| SRC | SRC proto-oncogene, non-receptor tyrosine kinase |  |
| GRB2 | growth factor receptor-bound protein 2 |  |
| P2RY1 | purinergic receptor P2Y, G protein coupled, 1 |  |
| GNA13 | guanine nucleotide binding protein (G protein), alpha 13 |  |
| FN1 | fibronectin 1 |  |
| PLEK | pleckstrin |  |
| PRKCA | protein kinase C, alpha |  |
| GP9 | glycoprotein IX (platelet) |  |
| PLA2G4A | phospholipase A2, group IVA (cytosolic, calcium-dependent) |  |
| TREML1 | triggering receptor expressed on myeloid cells-like 1 |  |
| CAP1 | CAP, adenylate cyclase-associated protein 1 (yeast) |  |
| COL3A1 | collagen, type III, alpha 1 |  |
| F8A1 | coagulation factor VIII-associated 1 |  |
| PRKCH | protein kinase C, eta |  |
| SHC1 | SHC (Src homology 2 domain containing) transforming protein 1 |  |
| GNB1 | guanine nucleotide binding protein (G protein), beta polypeptide 1 |  |
| FLNA | filamin A, alpha |  |
| LCK | LCK proto-oncogene, Src family tyrosine kinase |  |
| CLU | clusterin |  |
| APOA1 | apolipoprotein A-I |  |
| TMSB4X | thymosin beta 4, X-linked |  |
| FYN | FYN proto-oncogene, Src family tyrosine kinase |  |
| TYRO3 | TYRO3 protein tyrosine kinase |  |
| ABCC4 | ATP-binding cassette, subfamily C (CFTR/MRP), member 4 |  |
| VEGFA | vascular endothelial growth factor A |  |
| LCP2 | lymphocyte cytosolic protein 2 (SH2 domain containing leukocyte protein of 76kDa) |  |
| VCL | vinculin |  |
| F2R | coagulation factor II (thrombin) receptor |  |
| DGKB | diacylglycerol kinase, beta 90kDa |  |
| ADRA2B | adrenoceptor alpha 2B |  |
| PSAP | prosaposin |  |
| AKT1 | v-akt murine thymoma viral oncogene homolog 1 |  |
| BRPF3 | bromodomain and PHD finger containing, 3 |  |
| MPL | MPL proto-oncogene, thrombopoietin receptor |  |
| ITPR2 | inositol 1,4,5-trisphosphate receptor, type 2 |  |
| PDGFB | platelet-derived growth factor beta polypeptide |  |
| ITGA2B | integrin, alpha 2b (platelet glycoprotein IIb of IIb/IIIa complex, antigen CD41) |  |
| ARRB1 | arrestin, beta 1 |  |
| ACTN4 | actinin, alpha 4 |  |
| POTEM | POTE ankyrin domain family, member M |  |
| GNAI2 | guanine nucleotide binding protein (G protein), alpha inhibiting activity polypeptide 2 |  |
| CALM1 | calmodulin 1 (phosphorylase kinase, delta) |  |
| PRKCG | protein kinase C, gamma |  |
| DAGLB | diacylglycerol lipase, beta |  |
| MGLL | monoglyceride lipase |  |
| F2RL3 | coagulation factor II (thrombin) receptor-like 3 |  |
| VEGFB | vascular endothelial growth factor B |  |
| IGF1 | insulin-like growth factor 1 (somatomedin C) |  |
| IL6 | interleukin 6 |  |
| GAS6 | growth arrest-specific 6 |  |
| CALM3 | calmodulin 3 (phosphorylase kinase, delta) |  |
| VAV2 | vav 2 guanine nucleotide exchange factor |  |
| ADRA2C | adrenoceptor alpha 2C |  |
| CD63 | CD63 molecule |  |
| LAT | linker for activation of T cells |  |
| F8 | coagulation factor VIII, procoagulant component |  |
| ADAMTS13 | ADAM metallopeptidase with thrombospondin type 1 motif, 13 |  |
| SCG3 | secretogranin III |  |
| SRF | serum response factor (c-fos serum response element-binding transcription factor) |  |
| WNT3A | wingless-type MMTV integration site family, member 3A |  |
| PIK3CG | phosphatidylinositol-4,5-bisphosphate 3-kinase, catalytic subunit gamma |  |
| PLCG2 | phospholipase C, gamma 2 (phosphatidylinositol-specific) |  |
| RAC2 | ras-related C3 botulinum toxin substrate 2 (rho family, small GTP binding protein Rac2) |  |
| GNA12 | guanine nucleotide binding protein (G protein) alpha 12 |  |
| RAC1 | ras-related C3 botulinum toxin substrate 1 (rho family, small GTP binding protein Rac1) |  |
| CALU | calumenin |  |
| GNA15 | guanine nucleotide binding protein (G protein), alpha 15 (Gq class) |  |
| WDR1 | WD repeat domain 1 |  |
| DGKQ | diacylglycerol kinase, theta 110kDa |  |
| SELP | selectin P (granule membrane protein 140kDa, antigen CD62) |  |
| VEGFC | vascular endothelial growth factor C |  |
| SERPINA1 | serpin peptidase inhibitor, clade A (alpha-1 antiproteinase, antitrypsin), member 1 |  |
| THPO | thrombopoietin |  |
| HSPA5 | heat shock 70kDa protein 5 (glucose-regulated protein, 78kDa) |  |
| TRPC7 | transient receptor potential cation channel, subfamily C, member 7 |  |
| PIK3R1 | phosphoinositide-3-kinase, regulatory subunit 1 (alpha) |  |
| FGB | fibrinogen beta chain |  |
| ITGB3 | integrin, beta 3 (platelet glycoprotein IIIa, antigen CD61) |  |
| BLOC1S3 | biogenesis of lysosomal organelles complex-1, subunit 3 |  |
| RAPGEF3 | Rap guanine nucleotide exchange factor (GEF) 3 |  |
| RAP1A | RAP1A, member of RAS oncogene family |  |
| GP5 | glycoprotein V (platelet) |  |
| CFD | complement factor D (adipsin) |  |
| PRKCB | protein kinase C, beta |  |
| PECAM1 | platelet/endothelial cell adhesion molecule 1 |  |
| PLG | plasminogen |  |
| AXL | AXL receptor tyrosine kinase |  |
| SERPINE1 | serpin peptidase inhibitor, clade E (nexin, plasminogen activator inhibitor type 1), member 1 |  |
| CX3CL1 | chemokine (C-X3-C motif) ligand 1 |  |
| TUBA4A | tubulin, alpha 4a |  |

**Table S4.** Differentially expressed targeted mRNAs associated with blood coagulation

| **Gene symbol** | **Protein Name** |
| --- | --- |
| LYN | LYN proto-oncogene, Src family tyrosine kinase |
| GGCX | gamma-glutamyl carboxylase |
| PDE9A | phosphodiesterase 9A |
| SIRPA | signal-regulatory protein alpha |
| MMRN1 | multimerin 1 |
| EFEMP2 | EGF containing fibulin-like extracellular matrix protein 2 |
| HPS1 | Hermansky-Pudlak syndrome 1 |
| VWF | von Willebrand factor |
| RAF1 | Raf-1 proto-oncogene, serine/threonine kinase |
| KIF9 | kinesin family member 9 |
| KDM1A | lysine (K)-specific demethylase 1A |
| ENTPD1 | ectonucleoside triphosphate diphosphohydrolase 1 |
| LRRC16A | leucine-rich repeat containing 16A |
| ANO6 | anoctamin 6 |
| ITGAV | integrin, alpha V |
| CDC42 | cell division cycle 42 |
| RASGRP1 | RAS guanyl releasing protein 1 (calcium and DAG-regulated) |
| PFN1 | profilin 1 |
| SELPLG | selectin P ligand |
| FGR | FGR proto-oncogene, Src family tyrosine kinase |
| HDAC1 | histone deacetylase 1 |
| EGF | epidermal growth factor |
| KIF23 | kinesin family member 23 |
| DGKH | diacylglycerol kinase, eta |
| AKAP1 | A kinase (PRKA) anchor protein 1 |
| ITGA1 | integrin, alpha 1 |
| EHD2 | EH-domain containing 2 |
| MAPK14 | mitogen-activated protein kinase 14 |
| ITPK1 | inositol-tetrakisphosphate 1-kinase |
| CD59 | CD59 molecule, complement regulatory protein |
| GNAI3 | guanine nucleotide binding protein (G protein), alpha inhibiting activity polypeptide 3 |
| KRAS | Kirsten rat sarcoma viral oncogene homolog |
| EHD1 | EH-domain containing 1 |
| SERPING1 | serpin peptidase inhibitor, clade G (C1 inhibitor), member 1 |
| TBXA2R | thromboxane A2 receptor |
| IRF2 | interferon regulatory factor 2 |
| CDK2 | cyclin-dependent kinase 2 |
| IGF2 | insulin-like growth factor 2 |
| F13B | coagulation factor XIII, B polypeptide |
| CAV1 | caveolin 1, caveolae protein, 22kDa |
| MAG | myelin associated glycoprotein |
| PRKCD | protein kinase C, delta |
| RAPGEF4 | Rap guanine nucleotide exchange factor (GEF) 4 |
| TGFB1 | transforming growth factor, beta 1 |
| PRKAR1B | protein kinase, cAMP-dependent, regulatory, type I, beta |
| INPP5D | inositol polyphosphate-5-phosphatase, 145kDa |
| GP1BB | glycoprotein Ib (platelet), beta polypeptide |
| AKAP10 | A kinase (PRKA) anchor protein 10 |
| IFNB1 | interferon, beta 1, fibroblast |
| SPARC | secreted protein, acidic, cysteine-rich (osteonectin) |
| F5 | coagulation factor V (proaccelerin, labile factor) |
| P2RY12 | purinergic receptor P2Y, G protein coupled, 12 |
| MAPK1 | mitogen-activated protein kinase 1 |
| DOK2 | docking protein 2, 56kDa |
| HMCN1 | hemicentin 1 |
| NFE2 | nuclear factor, erythroid 2 |
| H3F3B | H3 histone, family 3B (H3.3B) |
| PRKACB | protein kinase, cAMP-dependent, catalytic, beta |
| ADORA2A | adenosine A2a receptor |
| PRKG1 | protein kinase, cGMP-dependent, type I |
| CAPZB | capping protein (actin filament) muscle Z-line, beta |
| JAM2 | junctional adhesion molecule 2 |
| LMAN1 | lectin, mannose-binding, 1 |
| PDE3A | phosphodiesterase 3A, cGMP-inhibited |
| APBB1IP | amyloid beta (A4) precursor protein-binding, family B, member 1 interacting protein |
| PPIA | peptidylprolyl isomerase A (cyclophilin A) |
| YWHAZ | tyrosine 3-monooxygenase/tryptophan 5-monooxygenase activation protein, zeta |
| KIF3A | kinesin family member 3A |
| CD9 | CD9 molecule |
| STIM1 | stromal interaction molecule 1 |
| SERPINA10 | serpin peptidase inhibitor, clade A (alpha-1 antiproteinase, antitrypsin), member 10 |
| PRTN3 | proteinase 3 |
| GNA11 | guanine nucleotide binding protein (G protein), alpha 11 (Gq class) |
| KIF11 | kinesin family member 11 |
| TEK | TEK tyrosine kinase, endothelial |
| CSK | c-src tyrosine kinase |
| ITGAM | integrin, alpha M (complement component 3 receptor 3 subunit) |
| SHH | sonic hedgehog |
| LYST | lysosomal trafficking regulator |
| ACTB | actin, beta |
| CFL1 | cofilin 1 (non-muscle) |
| CD2 | CD2 molecule |
| IRF1 | interferon regulatory factor 1 |
| ARRB2 | arrestin, beta 2 |
| NOS3 | nitric oxide synthase 3 (endothelial cell) |
| ATP2B2 | ATPase, Ca++ transporting, plasma membrane 2 |
| PRKG2 | protein kinase, cGMP-dependent, type II |
| PTK2 | protein tyrosine kinase 2 |
| BCAR1 | breast cancer anti-estrogen resistance 1 |
| SLC16A8 | solute carrier family 16 (monocarboxylate transporter), member 8 |
| GUCY1A2 | guanylate cyclase 1, soluble, alpha 2 |
| EHD3 | EH-domain containing 3 |
| RASGRP2 | RAS guanyl releasing protein 2 (calcium and DAG-regulated) |
| PIK3R5 | phosphoinositide-3-kinase, regulatory subunit 5 |
| F3 | coagulation factor III (thromboplastin, tissue factor) |
| RCOR1 | REST corepressor 1 |
| SLC8A1 | solute carrier family 8 (sodium/calcium exchanger), member 1 |
| PDE2A | phosphodiesterase 2A, cGMP-stimulated |
| RAB27A | RAB27A, member RAS oncogene family |
| ITGA10 | integrin, alpha 10 |
| PIK3CB | phosphatidylinositol-4,5-bisphosphate 3-kinase, catalytic subunit beta |
| LRP8 | low density lipoprotein receptor-related protein 8, apolipoprotein e receptor |
| ESAM | endothelial cell adhesion molecule |
| LAMP2 | lysosomal-associated membrane protein 2 |
| DAGLA | diacylglycerol lipase, alpha |
| GATA2 | GATA binding protein 2 |
| HGF | hepatocyte growth factor (hepapoietin A; scatter factor) |
| ACTN2 | actinin, alpha 2 |
| CRK | v-crk avian sarcoma virus CT10 oncogene homolog |
| DGKI | diacylglycerol kinase, iota |
| TLN1 | talin 1 |
| F2RL2 | coagulation factor II (thrombin) receptor-like 2 |
| OLR1 | oxidized low density lipoprotein (lectin-like) receptor 1 |
| CBX5 | chromobox homolog 5 |
| TGFB3 | transforming growth factor, beta 3 |
| PLAT | plasminogen activator, tissue |
| CD47 | CD47 molecule |
| APOB | apolipoprotein B |
| SIRPG | signal-regulatory protein gamma |
| SLC7A5 | solute carrier family 7 (amino acid transporter light chain, L system), member 5 |
| GRB7 | growth factor receptor-bound protein 7 |
| RHOG | ras homolog family member G |
| TGFB2 | transforming growth factor, beta 2 |
| PIK3R6 | phosphoinositide-3-kinase, regulatory subunit 6 |
| TRPC3 | transient receptor potential cation channel, subfamily C, member 3 |
| GATA6 | GATA binding protein 6 |
| DGKA | diacylglycerol kinase, alpha 80kDa |
| CD44 | CD44 molecule (Indian blood group) |
| HNF4A | hepatocyte nuclear factor 4, alpha |
| GNG2 | guanine nucleotide binding protein (G protein), gamma 2 |
| MFN2 | mitofusin 2 |
| ITGAL | integrin, alpha L (antigen CD11A (p180), lymphocyte function-associated antigen 1; alpha polypeptide) |
| TREM1 | triggering receptor expressed on myeloid cells 1 |
| GATA5 | GATA binding protein 5 |
| KCNMB2 | potassium large conductance calcium-activated channel, subfamily M, beta member 2 |
| ACTN1 | actinin, alpha 1 |
| ATP1B1 | ATPase, Na+/K+ transporting, beta 1 polypeptide |
| KLC1 | kinesin light chain 1 |
| DGKK | diacylglycerol kinase, kappa |
| PIK3CA | phosphatidylinositol-4,5-bisphosphate 3-kinase, catalytic subunit alpha |
| PAPSS2 | 3'-phosphoadenosine 5'-phosphosulfate synthase 2 |
| AP3B1 | adaptor-related protein complex 3, beta 1 subunit |
| ITPR3 | inositol 1,4,5-trisphosphate receptor, type 3 |
| GATA3 | GATA binding protein 3 |
| CD84 | CD84 molecule |
| KLC2 | kinesin light chain 2 |
| PDE1B | phosphodiesterase 1B, calmodulin-dependent |
| SYK | spleen tyrosine kinase |
| ITGA5 | integrin, alpha 5 (fibronectin receptor, alpha polypeptide) |
| PTPN1 | protein tyrosine phosphatase, non-receptor type 1 |
| SOS1 | son of sevenless homolog 1 (Drosophila) |
| PDPK1 | 3-phosphoinositide dependent protein kinase 1 |
| F2RL1 | coagulation factor II (thrombin) receptor-like 1 |
| CALM2 | calmodulin 2 (phosphorylase kinase, delta) |
| CDK5 | cyclin-dependent kinase 5 |
| PRKCQ | protein kinase C, theta |
| CYP4F2 | cytochrome P450, family 4, subfamily F, polypeptide 2 |
| ATP2B4 | ATPase, Ca++ transporting, plasma membrane 4 |
| RHOB | ras homolog family member B |
| HMG20B | high mobility group 20B |
| SERPINF2 | serpin peptidase inhibitor, clade F (alpha-2 antiplasmin, pigment epithelium derived factor), member 2 |
| SLC7A6 | solute carrier family 7 (amino acid transporter light chain, y+L system), member 6 |
| VKORC1 | vitamin K epoxide reductase complex, subunit 1 |
| JAM3 | junctional adhesion molecule 3 |
| KCNMB4 | potassium large conductance calcium-activated channel, subfamily M, beta member 4 |
| ANXA8 | annexin A8 |
| COL1A1 | collagen, type I, alpha 1 |
| MAFK | v-maf avian musculoaponeurotic fibrosarcoma oncogene homolog K |
| PRKCZ | protein kinase C, zeta |
| DOCK9 | dedicator of cytokinesis 9 |
| SLC8A3 | solute carrier family 8 (sodium/calcium exchanger), member 3 |
| SERPINA5 | serpin peptidase inhibitor, clade A (alpha-1 antiproteinase, antitrypsin), member 5 |
| C6orf25 | chromosome 6 open reading frame 25 |
| KIF2C | kinesin family member 2C |
| LEFTY2 | left-right determination factor 2 |
| VAV3 | vav 3 guanine nucleotide exchange factor |
| PLAUR | plasminogen activator, urokinase receptor |
| GATA4 | GATA binding protein 4 |
| KNG1 | kininogen 1 |
| PRKAR1A | protein kinase, cAMP-dependent, regulatory, type I, alpha |
| RAD51B | RAD51 paralog B |
| KIAA1715 | KIAA1715 |
| DGKG | diacylglycerol kinase, gamma 90kDa |
| RHOA | ras homolog family member A |
| FCER1G | Fc fragment of IgE, high affinity I, receptor for; gamma polypeptide |
| ADRA2A | adrenoceptor alpha 2A |
| ITPR1 | inositol 1,4,5-trisphosphate receptor, type 1 |
| THBS1 | thrombospondin 1 |
| KCNMA1 | potassium large conductance calcium-activated channel, subfamily M, alpha member 1 |
| DOCK1 | dedicator of cytokinesis 1 |
| GNAQ | guanine nucleotide binding protein (G protein), q polypeptide |
| TIMP1 | TIMP metallopeptidase inhibitor 1 |
| VAV1 | vav 1 guanine nucleotide exchange factor |
| PDE5A | phosphodiesterase 5A, cGMP-specific |
| PLAU | plasminogen activator, urokinase |
| GNA14 | guanine nucleotide binding protein (G protein), alpha 14 |
| GNAT3 | guanine nucleotide binding protein, alpha transducing 3 |
| ATP2A3 | ATPase, Ca++ transporting, ubiquitous |
| SRC | SRC proto-oncogene, non-receptor tyrosine kinase |
| GRB2 | growth factor receptor-bound protein 2 |
| P2RY1 | purinergic receptor P2Y, G protein coupled, 1 |
| CXADR | coxsackie virus and adenovirus receptor |
| PDE1A | phosphodiesterase 1A, calmodulin-dependent |
| GNA13 | guanine nucleotide binding protein (G protein), alpha 13 |
| FN1 | fibronectin 1 |
| PLEK | pleckstrin |
| PRKCA | protein kinase C, alpha |
| ITGB2 | integrin, beta 2 (complement component 3 receptor 3 and 4 subunit) |
| GP9 | glycoprotein IX (platelet) |
| PLA2G4A | phospholipase A2, group IVA (cytosolic, calcium-dependent) |
| ABL1 | ABL proto-oncogene 1, non-receptor tyrosine kinase |
| CAP1 | CAP, adenylate cyclase-associated protein 1 (yeast) |
| PRKAR2B | protein kinase, cAMP-dependent, regulatory, type II, beta |
| CAPZA1 | capping protein (actin filament) muscle Z-line, alpha 1 |
| AK3 | adenylate kinase 3 |
| PRKCH | protein kinase C, eta |
| TSPAN32 | tetraspanin 32 |
| SHC1 | SHC (Src homology 2 domain containing) transforming protein 1 |
| GNB1 | guanine nucleotide binding protein (G protein), beta polypeptide 1 |
| ANGPT2 | angiopoietin 2 |
| FLNA | filamin A, alpha |
| LCK | LCK proto-oncogene, Src family tyrosine kinase |
| SLC7A8 | solute carrier family 7 (amino acid transporter light chain, L system), member 8 |
| CLU | clusterin |
| KCNMB1 | potassium large conductance calcium-activated channel, subfamily M, beta member 1 |
| PTPN11 | protein tyrosine phosphatase, non-receptor type 11 |
| APOA1 | apolipoprotein A-I |
| KIF5A | kinesin family member 5A |
| DOCK6 | dedicator of cytokinesis 6 |
| TMSB4X | thymosin beta 4, X-linked |
| C4BPB | complement component 4 binding protein, beta |
| YES1 | YES proto-oncogene 1, Src family tyrosine kinase |
| ZFPM1 | zinc finger protein, FOG family member 1 |
| CD177 | CD177 molecule |
| FYN | FYN proto-oncogene, Src family tyrosine kinase |
| PLCG1 | phospholipase C, gamma 1 |
| SELE | selectin E |
| ITGA3 | integrin, alpha 3 (antigen CD49C, alpha 3 subunit of VLA-3 receptor) |
| WAS | Wiskott-Aldrich syndrome |
| KIF15 | kinesin family member 15 |
| SLC16A1 | solute carrier family 16 (monocarboxylate transporter), member 1 |
| CABLES1 | Cdk5 and Abl enzyme substrate 1 |
| THBD | thrombomodulin |
| ABCC4 | ATP-binding cassette, subfamily C (CFTR/MRP), member 4 |
| KIF2A | kinesin heavy chain member 2A |
| MMP1 | matrix metallopeptidase 1 (interstitial collagenase) |
| VEGFA | vascular endothelial growth factor A |
| LCP2 | lymphocyte cytosolic protein 2 (SH2 domain containing leukocyte protein of 76kDa) |
| ATP1B3 | ATPase, Na+/K+ transporting, beta 3 polypeptide |
| MAFG | v-maf avian musculoaponeurotic fibrosarcoma oncogene homolog G |
| VCL | vinculin |
| RACGAP1 | Rac GTPase activating protein 1 |
| ATP1B2 | ATPase, Na+/K+ transporting, beta 2 polypeptide |
| F2R | coagulation factor II (thrombin) receptor |
| ATP2A2 | ATPase, Ca++ transporting, cardiac muscle, slow twitch 2 |
| DGKB | diacylglycerol kinase, beta 90kDa |
| ADRA2B | adrenoceptor alpha 2B |
| SCUBE1 | signal peptide, CUB domain, EGF-like 1 |
| KIF3C | kinesin family member 3C |
| PTPRJ | protein tyrosine phosphatase, receptor type, J |
| PSAP | prosaposin |
| AKT1 | v-akt murine thymoma viral oncogene homolog 1 |
| BRPF3 | bromodomain and PHD finger containing, 3 |
| RBSN | zinc finger, FYVE domain containing 20 |
| MPL | MPL proto-oncogene, thrombopoietin receptor |
| HPS5 | Hermansky-Pudlak syndrome 5 |
| ITPR2 | inositol 1,4,5-trisphosphate receptor, type 2 |
| CD48 | CD48 molecule |
| ITGA4 | integrin, alpha 4 (antigen CD49D, alpha 4 subunit of VLA-4 receptor) |
| PDGFB | platelet-derived growth factor beta polypeptide |
| ORAI1 | ORAI calcium release-activated calcium modulator 1 |
| GATA1 | GATA binding protein 1 (globin transcription factor 1) |
| PRKAR2A | protein kinase, cAMP-dependent, regulatory, type II, alpha |
| ITGA2B | integrin, alpha 2b (platelet glycoprotein IIb of IIb/IIIa complex, antigen CD41) |
| ARRB1 | arrestin, beta 1 |
| ACTN4 | actinin, alpha 4 |
| GUCY1A3 | guanylate cyclase 1, soluble, alpha 3 |
| SELL | selectin L |
| VPS45 | vacuolar protein sorting 45 homolog (S. cerevisiae) |
| POTEM | POTE ankyrin domain family, member M |
| GNAI2 | guanine nucleotide binding protein (G protein), alpha inhibiting activity polypeptide 2 |
| CALM1 | calmodulin 1 (phosphorylase kinase, delta) |
| PRKCG | protein kinase C, gamma |
| KIF4B | kinesin family member 4B |
| F11 | coagulation factor XI |
| DAGLB | diacylglycerol lipase, beta |
| MGLL | monoglyceride lipase |
| ITGAX | integrin, alpha X (complement component 3 receptor 4 subunit) |
| SH2B1 | SH2B adaptor protein 1 |
| ITGA6 | integrin, alpha 6 |
| F2RL3 | coagulation factor II (thrombin) receptor-like 3 |
| PRCP | prolylcarboxypeptidase (angiotensinase C) |
| VEGFB | vascular endothelial growth factor B |
| IGF1 | insulin-like growth factor 1 (somatomedin C) |
| GAS6 | growth arrest-specific 6 |
| CALM3 | calmodulin 3 (phosphorylase kinase, delta) |
| VAV2 | vav 2 guanine nucleotide exchange factor |
| CEACAM8 | carcinoembryonic antigen-related cell adhesion molecule 8 |
| PTGIR | prostaglandin I2 (prostacyclin) receptor (IP) |
| ADRA2C | adrenoceptor alpha 2C |
| CD63 | CD63 molecule |
| LAT | linker for activation of T cells |
| SLC7A10 | solute carrier family 7 (neutral amino acid transporter light chain, asc system), member 10 |
| ATP2B1 | ATPase, Ca++ transporting, plasma membrane 1 |
| F11R | F11 receptor |
| PTPN6 | protein tyrosine phosphatase, non-receptor type 6 |
| NRAS | neuroblastoma RAS viral (v-ras) oncogene homolog |
| F8 | coagulation factor VIII, procoagulant component |
| ADAMTS13 | ADAM metallopeptidase with thrombospondin type 1 motif, 13 |
| SLC8A2 | solute carrier family 8 (sodium/calcium exchanger), member 2 |
| NOS1 | nitric oxide synthase 1 (neuronal) |
| SCG3 | secretogranin III |
| PPIL2 | peptidylprolyl isomerase (cyclophilin)-like 2 |
| MRVI1 | murine retrovirus integration site 1 homolog |
| SH2B2 | SH2B adaptor protein 2 |
| NBEAL2 | neurobeachin-like 2 |
| ATP2A1 | ATPase, Ca++ transporting, cardiac muscle, fast twitch 1 |
| HRAS | Harvey rat sarcoma viral oncogene homolog |
| PDE11A | phosphodiesterase 11A |
| PIK3CG | phosphatidylinositol-4,5-bisphosphate 3-kinase, catalytic subunit gamma |
| SIN3A | SIN3 transcription regulator family member A |
| PRKACA | protein kinase, cAMP-dependent, catalytic, alpha |
| PLCG2 | phospholipase C, gamma 2 (phosphatidylinositol-specific) |
| ENPP4 | ectonucleotide pyrophosphatase/phosphodiesterase 4 (putative) |
| RAC2 | ras-related C3 botulinum toxin substrate 2 (rho family, small GTP binding protein Rac2) |
| GNA12 | guanine nucleotide binding protein (G protein) alpha 12 |
| MAFF | v-maf avian musculoaponeurotic fibrosarcoma oncogene homolog F |
| RAC1 | ras-related C3 botulinum toxin substrate 1 (rho family, small GTP binding protein Rac1) |
| CALU | calumenin |
| GNA15 | guanine nucleotide binding protein (G protein), alpha 15 (Gq class) |
| WDR1 | WD repeat domain 1 |
| CEACAM1 | carcinoembryonic antigen-related cell adhesion molecule 1 (biliary glycoprotein) |
| SLC7A11 | solute carrier family 7 (anionic amino acid transporter light chain, xc- system), member 11 |
| DGKQ | diacylglycerol kinase, theta 110kDa |
| SELP | selectin P (granule membrane protein 140kDa, antigen CD62) |
| KIF26A | kinesin family member 26A |
| VEGFC | vascular endothelial growth factor C |
| MYB | v-myb avian myeloblastosis viral oncogene homolog |
| ANGPT4 | angiopoietin 4 |
| SERPINA1 | serpin peptidase inhibitor, clade A (alpha-1 antiproteinase, antitrypsin), member 1 |
| CEACAM6 | carcinoembryonic antigen-related cell adhesion molecule 6 (non-specific cross-reacting antigen) |
| THPO | thrombopoietin |
| HSPA5 | heat shock 70 kDa protein 5 (glucose-regulated protein, 78 kDa) |
| TRPC7 | transient receptor potential cation channel, subfamily C, member 7 |
| GUCY1B3 | guanylate cyclase 1, soluble, beta 3 |
| PIK3R1 | phosphoinositide-3-kinase, regulatory subunit 1 (alpha) |
| IFNA8 | interferon, alpha 8 |
| TP53 | tumor protein p53 |
| KIFAP3 | kinesin-associated protein 3 |
| FBLN5 | fibulin 5 |
| L1CAM | L1 cell adhesion molecule |
| CYP4F11 | cytochrome P450, family 4, subfamily F, polypeptide 11 |
| BSG | basigin (Ok blood group) |
| FGB | fibrinogen beta chain |
| KLKB1 | kallikrein B, plasma (Fletcher factor) 1 |
| SLC16A3 | solute carrier family 16 (monocarboxylate transporter), member 3 |
| ITGB3 | integrin, beta 3 (platelet glycoprotein IIIa, antigen CD61) |
| BLOC1S3 | biogenesis of lysosomal organelles complex-1, subunit 3 |
| RAPGEF3 | Rap guanine nucleotide exchange factor (GEF) 3 |
| RAP1A | RAP1A, member of RAS oncogene family |
| TFPI | tissue factor pathway inhibitor (lipoprotein-associated coagulation inhibitor) |
| GP5 | glycoprotein V (platelet) |
| CFD | complement factor D (adipsin) |
| PRKCB | protein kinase C, beta |
| PECAM1 | platelet/endothelial cell adhesion molecule 1 |
| F7 | coagulation factor VII (serum prothrombin conversion accelerator) |
| NOS2 | nitric oxide synthase 2, inducible |
| PLG | plasminogen |
| CD58 | CD58 molecule |
| KIF4A | kinesin family member 4A |
| SERPINE1 | serpin peptidase inhibitor, clade E (nexin, plasminogen activator inhibitor type 1), member 1 |
| HPS6 | Hermansky-Pudlak syndrome 6 |
| SPN | sialophorin |
| SH2B3 | SH2B adaptor protein 3 |
| TUBA4A | tubulin, alpha 4a |
